# Recommended Tool Compounds: Isoform- and Class-Specific Histone Deacetylase Inhibitors

**DOI:** 10.1021/acsptsci.5c00619

**Published:** 2026-02-20

**Authors:** Linda Schäker-Hübner, Finn K. Hansen

**Affiliations:** Pharmaceutical Institute, 9374 University of Bonn, An der Immenburg 4, Bonn 53121, Germany

**Keywords:** histone deacetylase (HDAC), HDAC inhibitor, HDAC assay, tool compound, chemical probe

## Abstract

The development of selective histone deacetylase inhibitors (HDACi) and thus the identification of suitable chemical probes is still highly complex. Moreover, in the past, the results of oversimplified biochemical HDAC assays have often been overinterpreted. This has led to the extensive use of various supposedly isoform-selective HDACi as chemical probes to investigate the biological function of specific HDAC isoforms and classes. Considering more recent insights concerning the structure, binding kinetics, as well as substrate specificity of several HDAC isoforms, at least some of these studies should be reevaluated thoroughly. In this review, we present a selection of possible tool compounds for use in biological and pharmacological studies to investigate the biological function of specific HDAC isoforms or classes and comprehensively discuss their limitations based on the currently available data.

## Graphical abstract

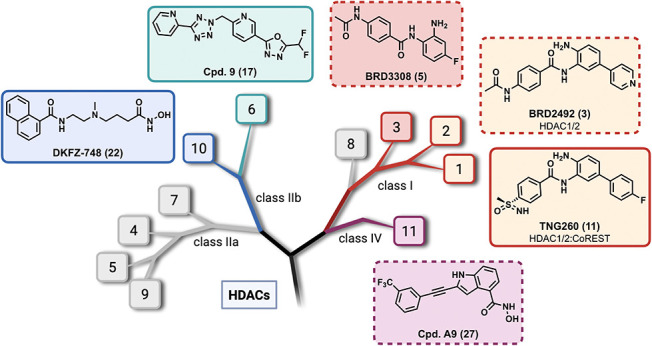

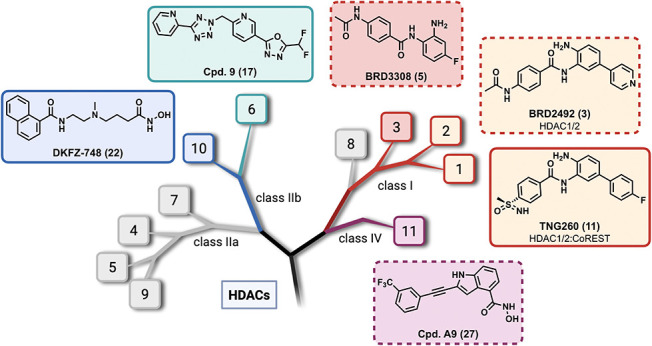

## Introduction

Histone deacetylases (HDACs) are a class of enzymes, which among others remove acetyl groups from ε-*N*-acetylated lysine side chains of histone and nonhistone proteins.^1,2^ Thus, far, 18 human HDACs have been identified.^1–3^ Based on their sequence similarities with yeast HDACs, they are categorized into four classes: class I (HDAC1, 2, 3, and 8), class II (HDAC4, 5, 7, 9, 6, and 10), class III (SIRT1–7), and class IV (HDAC11). Additionally, class II enzymes are further divided into two subclasses: class IIa (HDAC4, 5, 7, and 9) and class IIb (HDAC6 and 10).^1,4–6^ (see Figure 1) Furthermore, HDACs can be grouped into two protein families: the zinc-dependent HDAC protein family (class I, II, and IV) and the sirtuin protein family (class III).^7^ In contrast to zinc-dependent HDACs, the so-called sirtuins are NAD^+^-dependent protein deacylases or ADP-ribosyltransferases. By targeting a wide range of cellular proteins located in cytoplasm, mitochondria, and nucleus, sirtuins regulate gene expression and act as “metabolic sensors” in response to the cellular redox status.^5,6,8^ However, although both zinc-dependent HDACs and sirtuins remove acetyl groups from histone and nonhistone proteins, the abbreviation “HDAC” usually only refers to the zinc-dependent HDAC isoforms which are also the focus of this Review.

**Figure 1:**
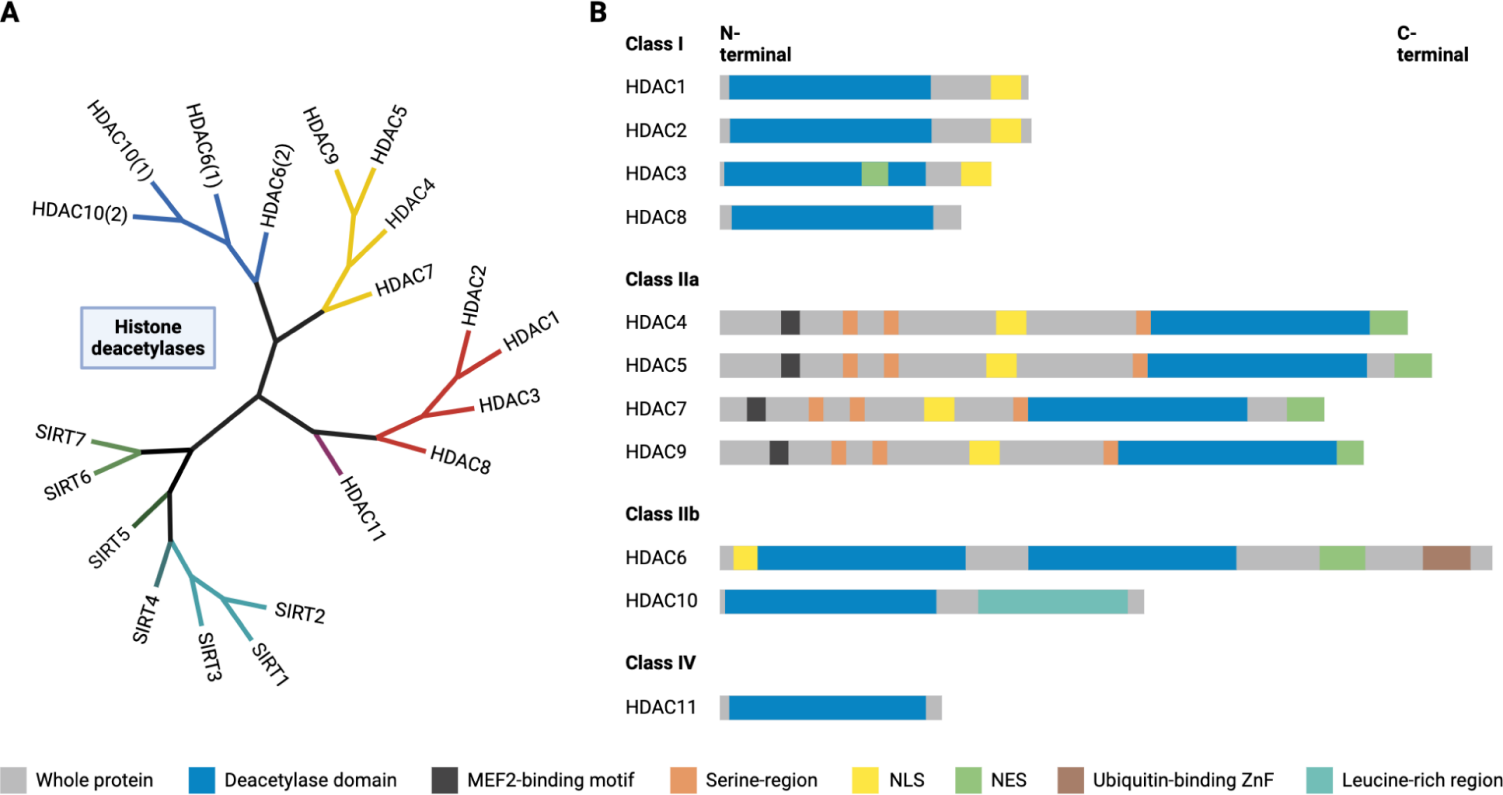
Phylogenetic tree and basic domain structure of the human histone deacetylase protein family. **A)** Human zinc-dependent HDACs and sirtuins are clustered on branches based on their amino acid sequences.^1,3,9,10^ **B)** Fundamental domain structure of human zinc-dependent HDACs.^5–7,11–13^ Myocyte enhancer factor 2 (MEF2); “Serine-region”: protein 14–3–3 binding motif; Nuclear export sequence (NES); nuclear localization sequence (NLS); Zinc finger (ZnF). Created in BioRender. Schäker-Hübner, L. (2025) https://BioRender.com/54yzzv0.

As mentioned above, the remaining 11 HDAC isoforms of class I, class II, and class IV are zinc-dependent amidohydrolases that catalyze the cleavage of amide bonds using water as nucleophile.^1,2^ Their substrate spectrum includes a wide array of post-translational modifications (PTMs) such as lysine formylation, acetylation, propionylation, crotonylation, butyrylation, lactylation, succinylation, myristoylation and palmitoylation.^2,14,15^ That way, HDACs mediate lysine deacylation of histones and various nonhistone proteins including α-tubulin, tau, p53, Hsp90, chromatin assembly factor 1 (CAF-1), estrogen-related receptor alpha (ERRα), structural maintenance of chromosomes protein 3 (SMC3), as well as many others.^1,2,16^

Since their discovery in 1996,^17^ HDACs have been implicated in a wide range of human diseases including numerous cancers,^18–30^ Alzheimer’s disease,^31^ Parkinson’s disease,^32^ Huntington’s disease,^33^ Friedreich’s ataxia,^34,35^ spinal muscular atrophy,^36^ Duchenne muscular dystrophy,^37^ idiopathic pulmonary fibrosis,^38^ and HIV infection.^39^ Consequently, HDACs are highly attractive epigenetic drug targets as well as an interesting research area that promises to provide invaluable insights into the pathogenesis and pathophysiology of several human diseases.

### Structural Characteristics of Zinc-Dependent HDAC Isoforms

Although the size of the different HDAC isoforms varies from 347 amino acids (HDAC11) to 1215 amino acids (HDAC6), all HDACs share a highly conserved deacetylase domain.^1,6^ (see Figure 1) Furthermore, especially within the different HDAC classes, several HDAC isoforms show similar expression patterns, high sequence homology, as well as deacetylase activity toward common substrates, suggesting functional redundancy among these HDACs in vivo.^7,11^ However, knockout experiments in mice showed that loss of function of any HDAC isoform either results in pre- or early post-natal death, or at least leads to a distinct (patho)-physiological phenotype thus demonstrating the unique role of each HDAC in development and survival.^11,40^

### Class I HDACs (HDAC1, 2, 3, and 8)

The class I isoforms HDAC1–3 operate as constituents of multiprotein complexes. As such, their functions depend on the structure and composition of the respective corepressor complexes.^6,41^ Additionally, the catalytic activity of HDAC1–3 in multiprotein complexes is stabilized by inositol phosphates, which serve as “intermolecular glue” between the respective HDAC isoform and corepressor proteins. The resulting protein–protein interaction presumably leads to an allosteric activation of class I HDACs in the respective multiprotein complex.^42,43^ Furthermore, HDAC1–3 possess an approximately 14 Å wide, lipophilic internal cavity adjacent to the catalytic zinc ion, the so-called “foot-pocket”.^1^ It is presumably used for water entry and/or the accommodation of acyl-groups with chain lengths longer than acetyl.^1,44^ The respective isoforms differ from each other by shape and size of this cavity that consequently presents an attractive target structure for the design of isoform selective HDAC inhibitors (HDACi).

Especially, the class I isoforms HDAC1 and HDAC2 share high sequence homology. HDAC1 and HDAC2 are usually coexpressed and interact with each other. Their homo- and heterodimers provide the catalytic cores of several large corepressor complexes, including repressor element-1 silencing transcription factor corepressor (CoREST), Mi-2/nucleosome remodeling deacetylase (NuRD), switch-independent-3A (Sin3A), and mitotic deacetylase complex (MiDAC).^2,6,41^ Besides their pronounced histone deacetylase activity as such, HDAC1 and HDAC2 target many other nuclear proteins involved in chromatin formation, DNA damage repair, DNA methylation, and transcriptional regulation including p53, CAF-1, and DNMT.^1,2^ For example, HDAC1 and HDAC2 are extensively involved in hematopoiesis. Their functions include lineage commitment determination, cell differentiation, and proliferation.^29^

In contrast to HDAC1 and HDAC2, which are strictly nuclear enzymes, HDAC3 is localized in both nucleus and cytoplasm.^13,45–47^ The subcellular localization of HDAC3 is mediated through a nuclear localization sequence (NLS) and an unusual nuclear export signal (NES) in the central portion of the protein (see Figure 1).^13^ HDAC3 is also distinguished from HDAC1 and HDAC2 by its C-terminal region which provides an extended binding domain for nuclear complexes.^2^ Interestingly, HDAC3 exclusively associates with only two nuclear receptor corepressors: the nuclear receptor corepressor 1 (NCoR1) and the silencing mediator of retinoic acid and thyroid hormone receptor (SMRT, NCoR2) which are essential for the catalytic function of HDAC3 (see “*In vitro* profiling of HDACi” below).^2,45,48^ Additionally, HDAC3 differs from HDAC1 and 2 concerning the size of its “foot pocket”. Due to a serine (in HDAC1/2) to tyrosine (HDAC3) switch at the bottom of the “foot pocket”, the “foot pocket” of HDAC3 is slightly smaller. This small structural difference is considered as an attractive target for the design of isoform selective HDAC inhibitors.^49–52^ *In vivo*, HDAC3 regulates many physiological processes and performs several tissue-specific functions: Among others, HDAC3 seems to integrate signals from the circadian clock into metabolism via the circadian nuclear receptor REV-ERBα,^45,53^ controls the glucose-stimulated insulin secretion in pancreatic β-cells,^54^ is involved in the regulation of p53-dependent apoptosis,^55,56^ hematopoietic stem cell development,^29^ cardiac development,^45^ lung development^45^ as well as the development and remodeling of bones.^57,58^ Furthermore, HDAC3 is highly expressed in the adult brain and seems to control glial cell fate in neural progenitor cells of the central nervous system.^59^ Accordingly, HDAC3 is also intricately involved in neurodevelopment and -function as well as learning and memory.^45,59,60^

HDAC8, the smallest of the class I enzymes, is X-linked in humans, ubiquitously expressed, and localized in both nucleus and cytoplasm.^2,6,30^ Unlike HDAC1–3, HDAC8 acts independently from multiprotein complexes since it lacks the C-terminal protein domain that enables interaction with additional cofactors (see Figure 1). Thus far, only few nonhistone proteins have been validated as deacetylation substrates of HDAC8, namely the estrogen-related receptor alpha (ERRα), p53, and structural maintenance of chromosomes protein 3 (SMC3).^2,16^ A limiting factor for the understanding of HDAC8 is its overall low *in vitro* deacetylation activity at peptide substrates (see “*In vitro* profiling of HDACi” below).^16^ However, HDAC8 is able to cleave acyllysine peptides with acyl chains with up to 16 carbons which are very likely accommodated by an internal hydrophobic cavity adjacent to the active site that resembles the “foot pocket” of HDAC1–3 but differs in shape and size.^50,61,62^ Notably, the catalytic efficiencies of HDAC8 are multiple times higher for octanoyl-, dodecanoyl-, and myristoyllysine than for acetyllysine, suggesting a physiologically relevant fatty acid deacylation activity of HDAC8.^16^ Furthermore, HDAC8 features the so-called “side pocket”, a unique hydrophobic groove on the protein surface surrounding the active site.^50,61,62^

### Class IIa HDACs (HDAC4, 5, 7, and 9)

Class IIa HDACs are rather large proteins which shuttle between nucleus and cytoplasm.^2,7,12,41^ Unlike class I HDACs, class IIa HDACs possess an extended N-terminal domain which contains a NLS, several conserved serine residues, as well as a conserved binding site for the transcription factor myocyte enhancer factor 2 (MEF2) (see Figure 1).^6,11^ In addition, all class IIa enzymes possess a corresponding NES which is located at the C-terminal region.^2,6,11,12^ The subcellular localization of class IIa HDACs is determined by the reversible phosphorylation of conserved, N-terminal serine residues: Following kinase-dependent phosphorylation, the respective class IIa isoform shuttles from the nucleus to the cytoplasm.^6,11^ Conversely, dephosphorylation causes the nuclear (re)­localization of class IIa HDACs leading to transcriptional repression by the recruitment of HDAC3.^6^ As mentioned above, all class IIa HDACs possess a binding site for the transcription factor MEF2, an important regulator of cellular differentiation, embryonic development, and stress response.^63^ Upon phosphorylation-induced export into the cytoplasm, class IIa HDACs dissociate from MEF2.^6^ This allows the transcriptional coactivator histone acetyltransferase (HAT) p300 to associate with MEF2 via the HDAC binding site and triggers e.g., myogenic differentiation.^11,64–66^ This emphasizes the importance of reversible phosphorylation of class IIa HDACs as a key mechanism in tissue development.

Accordingly, class IIa HDACs show highly tissue specific expression patterns: HDAC4 is highly expressed in brain, skeletal muscle, and growth plates of the skeleton but is not expressed in liver or lung.^67,68^ HDAC7 is specifically expressed in the vascular endothelium during early embryogenesis where it acts as an essential regulator of blood vessel development.^69^ Moreover, HDAC7 is involved in T cell development, a vital part of normal hematopoiesis.^70^ Similarly, HDAC5 and HDAC9 are enriched in heart, muscles, and brain.^11^

Interestingly, class IIa HDACs show very weak (∼1000-fold less) catalytic activity especially compared to class I HDACs.^7^ The catalytic domains of class I, IIb, and IV HDACs contain several highly conserved key residues (e.g., histidine (H), aspartic acid (D), and tyrosine (Y)) which are necessary for catalytic activity.^6^ However, in class IIa HDACs the key tyrosine is replaced by a histidine which leads to an insufficient substrate activation resulting in the characteristically weak catalytic activity of class IIa HDACs.^2,6,11^ Thus far, there is strong evidence suggesting that class IIa HDACs act as scaffolding proteins *in vivo*, which recruit acetyllysine substrates to HDAC3-containing multiprotein complexes. In this case, HDAC3 would facilitate the actual deacetylation of the presented substrates, not the respective class IIa isoform.^2,71^ Besides the severely reduced catalytic activity of the class IIa HDACs, the tyrosine to histidine switch is also responsible for the formation of the so-called “lower pocket”, an internal cavity situated at the bottom of the substrate binding tunnel near the catalytic zinc ion. The cavity is formed due to the “outward” facing conformation of the “switched” histidine residue (H976 in HDAC4).^50,72,73^ Similar to the characteristic “foot pocket” of the class I isoforms HDAC1–3, the “lower pocket” is an attractive target structure for the design of class IIa selective HDACi.^50,72–75^ However, due to the high sequence homology of the class IIa HDAC isoforms in the immediate vicinity of the active site, the rational design of class IIa isoform selective HDACi proves to be very difficult.

### Class IIb HDACs (HDAC6 and 10)

Similar to class IIa HDACs, HDAC6 holds both a NLS and a corresponding NES. However, unlike class IIa HDACs (see above) and class I HDACs, which are mostly nuclear, HDAC6 is mainly an efficient cytoplasmic deacetylase, but has also been reported as a partially nuclear enzyme.^11,76^ With a C-terminal ubiquitin-binding zinc finger domain (ZnF) as well as two (functional) catalytic domains (CDs), HDAC6 is also the largest HDAC (see Figure 1).^1,77^ Several proteins have been identified as HDAC6 substrates, including the cytoskeletal proteins α-tubulin, cortactin, and tau.^2,11,78^ Deacetylation of α-tubulin and cortactin is aided by a microtubule-binding domain which enables HDAC6 to directly affect cytoskeleton structure and therefore cell mobility.^2^ The molecular chaperone Hsp90, whose activity is regulated by reversible acetylation, is another substrate of HDAC6. Hsp90 is involved in the structural maturation (“folding”), and degradation of proteins. HDAC6 directly stimulates Hsp90 activity through interaction with both catalytic domains as well as the ubiquitin-binding ZnF thus promoting the assembly of functional Hsp90 chaperone complexes.^79,80^ Further, HDAC6 recognizes ubiquitinated proteins *via* the ubiquitin-binding ZnF and directs these misfolded proteins to degradation by perinuclear aggresomes.^2,79^ Another HDAC6 substrate is Ku70, a nuclear factor that binds to DNA double-strand breaks. Deacetylated, cytoplasmatic Ku70 however binds the apoptosis regulator BAX, preventing BAX-induced apoptosis and HDAC6 loss of function was shown to trigger BAX-dependent cell death.^81^ As mentioned above, HDAC6 holds two catalytic domains.^2,82^ In 2016, Hai and Christianson demonstrated that both CD1 and CD2 exhibit lysine deacetylase activity, but with interesting differences in substrate specificity.^77,82^ Although the key catalytic residues are identical in both catalytic domains, direct comparison of the active site entrance of HDAC6 CD1 and CD2 reveals notable differences.^82^ The most striking difference is the “gatekeeper” lysine of HDAC6 CD1 which appears to promote specific interactions with acetyllysine substrates bearing a free α-carboxylate group.^2,82^ However, while several proteins such as α-tubulin and tau have been identified as *in vivo* substrates of CD2, the biological function of CD1 remains somewhat enigmatic.^2,82^

HDAC10, localized in both nucleus and cytoplasm, is characterized by a deacetylase domain, a catalytically inactive pseudodeacetylase domain, as well as a C-terminal leucine-rich domain.^2,6^ Among others HDAC10 is involved in DNA repair, immunoregulation, and notably autophagy.^2,83^ Interestingly, HDAC10 is not a lysine deacetylase per se, but an efficient polyamine deacetylase. This substrate specificity is mainly facilitated through a glutamate “gatekeeper” and an additional steric bulk opposite of the gatekeeper residue which further constricts the active site. This leads to a substrate preference for long, slender polyamine substrates while excluding bulky acetyllysine containing proteins.^83,84^ Function and metabolism of polyamines like spermidine is regulated by reversible acetylation and more recently, both HDAC10 and spermidine have been identified as mediators of autophagy.^83^ Autophagy is an important cell-physiologic response to cellular stress e.g., nutrient deficiency.^85^

### Class IV HDACs (HDAC11)

HDAC11, discovered in 2002, is the only member of HDAC class IV.^2,6^ Primarily localized in the nucleus, HDAC11 seems to be a transcriptional regulator of immunomodulation and shows weak deacetylase activity.^2^ However, like HDAC8 (see above), it efficiently cleaves acyllysine residues with longer chain lengths such as dodecanoyllysine and myristoyllysine indicating that HDAC11 primarily operates as a fatty acid protein deacylase.^6,86,87^ Interestingly, homology modeling additionally suggests that the long chain acyl groups are accommodated within an internal hydrophobic cavity in the same manner as in HDAC8.^2,86^

Taken together, all HDACs share a highly conserved deacetylase domain^1,6^ and structural differences between the isoforms are mostly found at the protein surface of the entrance of the active site (e.g., “gatekeeper residues”).^1,2,82–84,88^ Additionally, some HDAC classes and/or isoforms are also distinguished from each other by isoform specific subpockets. This includes the so-called “foot-pocket” which is characteristic for some class I isoforms as well as the so-called “lower pocket”, characteristic for class IIa HDACs.^1,2,44,50,72,73^ Collectively, these and other structural features determine the substrate specificity of the different HDAC isoforms and/or classes *in vivo* and provide attractive target structures for the design of class or isoform selective HDACi as well as chemical probes (seeTable [Table tbl1]).

**Table 1:** Summarized Known Key Features of Human HDAC Isoforms

**Class**	**Isoform**	**Subcellular Localization**	**Key Features**	***In Vivo* Substrates**
**I**	HDAC1	nucleus	usually coexpressed; homo- or heterodimers provide catalytic cores for several large corepressor complexes	histones, transcriptional regulators
	HDAC2			
	HDAC3	nucleus/cytoplasm	association to NCoR1 or SMRT is necessary for enzymatic activity	histones, transcriptional regulators
	HDAC8	nucleus/cytoplasm	X-linked, independent from multiprotein complexes, *in vitro* preference for C_8_, C_12_, and C_14_ acyl lysines	ERRα, p53, SMC3, acyl lysine residues
**IIa**	HDAC4	nucleus/cytoplasm	tissue-specific expression, MEF2 binding domain, shuttle between nucleus and cytoplasm depending on phosphorylation status; weak catalytic activity	none; recruit acyllysine substrates to HDAC3
	HDAC5			
	HDAC7			
	HDAC9			
**IIb**	HDAC6	cytoplasm	ubiquitin and microtubule binding domains; two independent catalytic domains, gatekeeper lysine of CD1 promotes preference for substrates bearing a free α-carboxylate group	α-tubulin, cortactin, tau, Hsp90, Ku70
	HDAC10	nucleus/cytoplasm	polyamine deacetylase; glutamate “gatekeeper”, narrow substrate channel	acetylpolyamines, e.g., *N* ^8^-acetylspermidine
**IV**	HDAC11	nucleus/cytoplasm	weak deacetylase activity, *in vitro* preference for C_12_, and C_14_ acyllysines	transcriptional regulators, longer chain acyllysines

### HDAC Pharmacophore Model

Considering the overall structure of HDAC enzymes, some basic characteristics for the design of potent HDACi have been identified: First, the overall shape of the inhibitor should mimic the interactions between the HDAC enzyme and its natural ε-*N*-acetyllysine substrate. Second, the inhibitor should coordinate the catalytic zinc ion.^2,89^ Additionally, inhibitor binding benefits from specific interactions with the enzyme surface in the vicinity of the active site.^2^ As early as 1997, Jung et al. proposed the first general pharmacophore model for HDACi consisting of three basic elements: a “zinc binding group” (ZBG), a linker region and a “cap”.^89^ Although very simple, this model is still widely used and accounts for the majority of known HDACi.^1,2^ The requirements of this pharmacophore model are perfectly met by trichostatin A, as illustrated in Figure 2A.^89^ Trichostatin A was reported in 1990 as the first potent and specific inhibitor of histone deacetylation.^2,90^ Since then, various HDACi have been developed, a few well-established representatives are pictured in Figure 2C: Although ricolinostat was originally highlighted as a HDAC6 selective HDACi, trichostatin A, vorinostat, panobinostat, and ricolinostat are essentially pan-HDACi.^2^ Despite this, all of them are commonly used for biological studies which is rather problematic since the biological effects of such nonspecific inhibitors arise from the combined modulation of several HDAC isoforms. Due to this lack of isoform selectivity, pan-HDACi—including the substances depicted in Figure 2—are generally unsuitable as tool compounds.^91^

**Figure 2:**
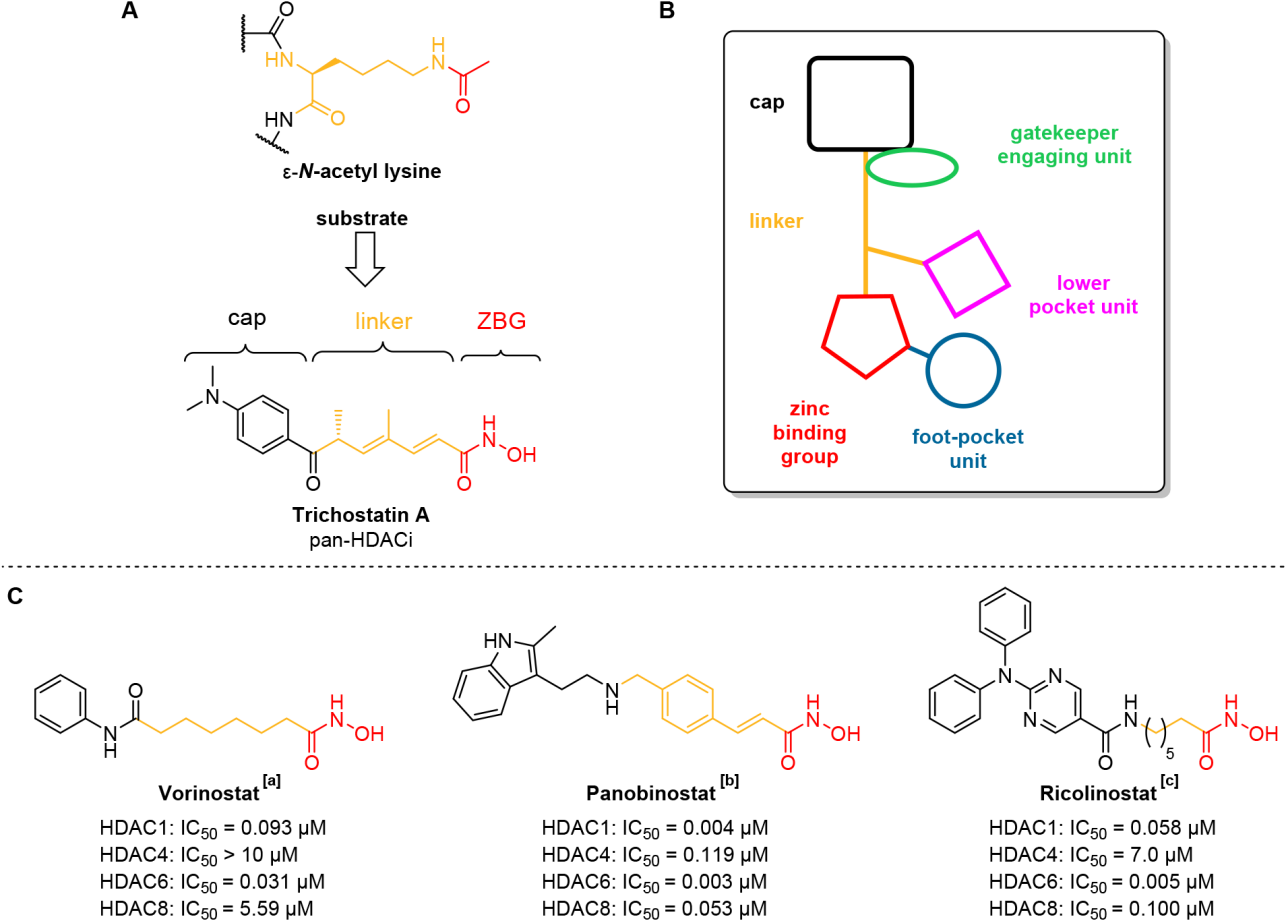
**A**) Simplified illustration of the natural peptidyl ε-*N*-acetyllysine substrate of most HDACs (top) in relation to the general pharmacophore model of HDACi proposed by Jung et al. using the example of vorinostat (bottom).^89^ **B**) Extended pharmacophore model for selective HDACi.^49,50,82,83,92^ **C**) Selected pan-HDACi. IC_50_ values are taken from the following references: [a] Kraft et al. 2024;^93^ [b] WO 2013/062344 A1;^94^ [c] Santo et al. 2012.^95^ Notably, reported isoform profile data result from different assay conditions and should therefore be interpreted with caution.

More recently, Sippl and coworkers^50^ as well as other researchers presented extended pharmacophore models of the HDAC binding pocket(s) which are useful for the design of selective HDACi. These extended models (e.g., see Figure 2B) take into account additional isoform specific subpockets that affect inhibitor selectivity toward certain HDAC classes and isoforms including the “foot-pocket” (class I),^49^ the “lower pocket” (class IIa)^72,92^ as well as the glutamate “gate keeper” (HDAC10).^83^

### *In Vitro* Profiling of HDAC Inhibition

A major aspect in the development of HDACi is the determination of HDAC inhibition *in vitro*. This is usually done using cell-free screening methods (hereafter called “HDAC assays”).^2^ Currently, most high-throughput HDAC assays are end-point assays which rely on the enzymatic activity of the enzymes: using acetyllysine derivatives similar to the N-terminal histone tail, the formation of deacetylated product is quantified. Typically, these artificial substrates hold an additional cleavable fluorophore at their C-terminus, which is used to indirectly determine product formation.^2,96,97^ The basic principle of such assays is illustrated in Figure 3.

**Figure 3:**
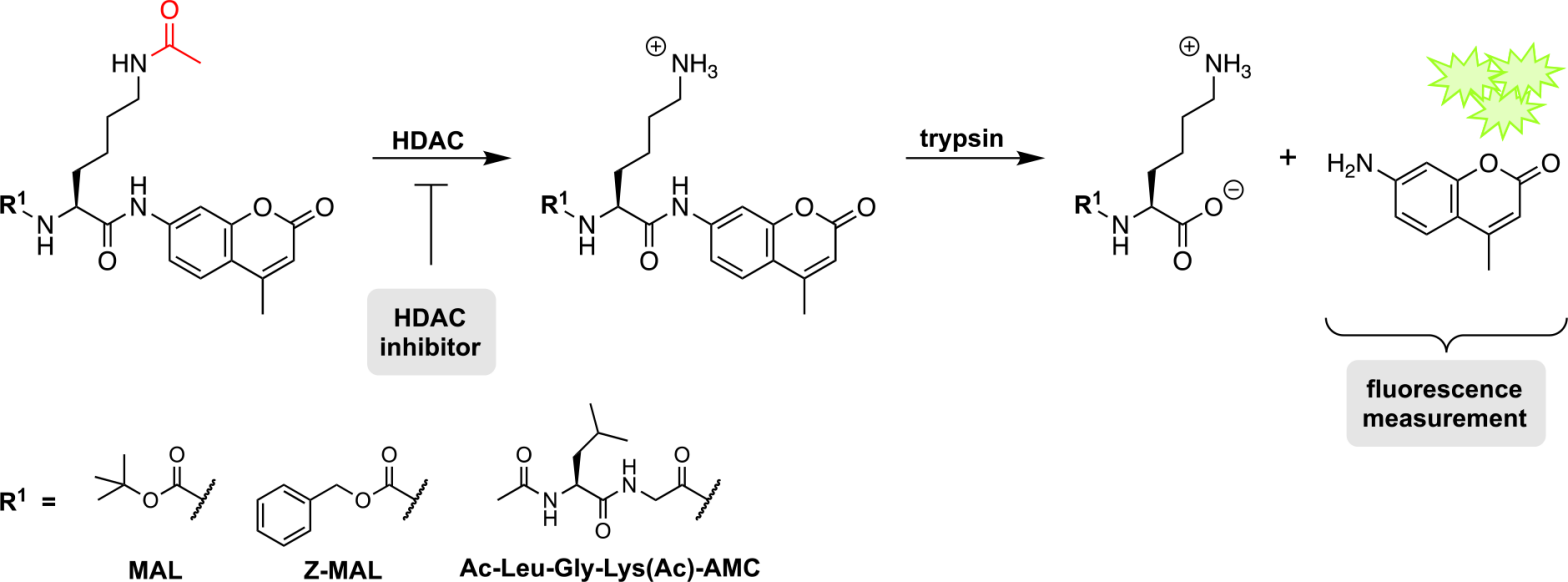
Basic sequence of fluorescence based *in vitro* HDAC assays based on enzymatic activity and product formation including some well-established fluorogenic substrates.^96,98^ Following the cleavage of the ε-*N*-acetyl-l-lysine, the C-terminal lysine is recognized by trypsin which subsequently cleaves the fluorophore. The resulting fluorescence signal is quantified and further used to generate inhibitor concentration–response curves.

First, the respective substrate undergoes acetyllysine hydrolysis catalyzed by an HDAC. In presence of an HDACi, this step gets less likely, leading to reduced HDAC activity and therefore less substrate conversion. Second, the resulting C-terminal lysine is recognized by trypsin or another suitable protease which is usually added after a certain incubation time. It cleaves the C-terminal amide quantitatively to release the fluorescent 7-amino-4-methylcoumarin (AMC).^2,96,98^ The typical readout to quantify the extend of enzyme inhibition is the half maximal inhibitory concentration (IC_50_) derived from the resulting inhibitor concentration–response curve.^99^

### Comparability of HDAC Inhibition Data

IC_50_ values are dependent on the specific assay setting (enzyme, substrate, substrate concentration, incubation time, and temperature etc.).^100^ Two basic problems originate from this fact: For one, IC_50_ values derived from different assays for the same HDAC isoform often vary immensely. Therefore, it is difficult to compare HDACi which were assessed in different HDAC assays. Related to this, it is also not easy to assess the selectivity profile of HDACi since different enzyme/substrate pairs are usually characterized by different Michaelis constants (*K*_M_).^100^ Therefore, the assessment of isoform selectivity among different enzymes should ideally rely on the comparison of inhibitor affinity which can be directly measured as dissociation constant (*K*_D_) or indirectly estimated from the IC_50_ as inhibition constant (*K*_i_) using the Cheng-Prusoff equation. However, calculating *K*_i_ from the respective IC_50_ requires the determination of *K*_M_ and is only applicable under certain conditions.^99–101^ Besides that, observed IC_50_ values can “shift” with varying enzyme–inhibitor preincubation times and depending on the chosen temperature (see section “Slow-Binding HDAC Inhibitors”).^102–105^

### Selection of Suitable Assay Conditions

In the past, HDACs where often obtained from cell extracts contain a mixture of mostly nuclear isoforms in an unknown ratio which severely limits the significance of the resulting data.^2^ By now, all HDAC isoforms are commercially available as recombinant enzymes. Using the substrates pictured in Figure 3, the human deacetylases HDAC1, HDAC2, HDAC6 (CD2),^82^ and HDAC3:NCoR2 (see above) show efficient substrate turnover *in vitro*. Therefore, *in vitro* HDAC inhibition data acquired for these isoforms seem to be valid in terms of isoform selectivity.^2,9^ In contrast, running HDAC assays against the remaining HDAC isoforms is still complicated in practice since they show very low deacetylase activity. This problem is usually “solved” by switching to more easily scissile trifluoroacetyllysine substrates leading to a higher substrate turnover.^2,9,98^

However, there are two basic issues with this solution: First, the class IIa enzymes HDAC4, 5, 7, and −9 are probably “reader” domains without relevant deacetylase activity *in vivo*.^2,71^ Interestingly, Bradner et al. were able to show that the substrates Z-MAL and Ac-Leu-Gly-Lys­(Ac)-AMC actually inhibit the conversion of Ac-Leu-Gly-Lys­(TFA)-AMC by class IIa isoforms *in vitro* demonstrating that acetyllysine substrates bind to class IIa enzymes but no chemical conversion takes place.^9^ Additionally, it has been reported that class IIa HDACs isolated from mammalian cells are occasionally contaminated with copurified class I HDACs. In these cases, the respective class I enzymes are largely responsible for the observed deacetylase activity.^106^ Consequently, the significance of assay results based on substrate conversion is severely limited and very likely does not correlate with HDAC class IIa isoform selectivity and function *in vivo*.^2^ Second, since HDAC8, and 11 are probably fatty acid deacylases^16,86,87^ and HDAC10 is most likely a polyamine deacetylase^83^ these enzymes do not function as lysine deacetylases *in vivo* either (see above). Consequently, trifluoroacetyllysine substrates are unsuitable for these isoforms as well and the significance of data derived from such assays is at least questionable.^2^ The development of new, more suitable substrates and/or assay methods (e.g., BRET, TR-FRET, microscale thermophoresis (MST), biolayer interferometry (BLI), and fluorescence polarization (FP))^107^ would immensely benefit HDACi development in all of these cases, since improved *in vitro* HDAC assays would likely provide more valid data in terms of predicting isoform selectivity *in vivo*. Fortunately, in recent years, several new HDAC assays for some of these isoforms have emerged (see below).

### Novel Assay Formats to Investigate the Polyamine Deacetylase HDAC10

In 2017, David W. Christianson and coworkers developed a discontinuous LCMS-based assay for HDAC10 to characterize steady-state kinetic parameters for the hydrolysis of a variety of nonfluorogenic acetylpolyamine substrates such as acetylputrescine and *N* ^8^-acetylspermidine.^83^ However, since this assay was not suitable for high-throughput screenings, Aubry K. Miller and coworkers developed two ligand displacement assays based on a newly developed tubastatin-Alexa647-tracer.^108^ Since both the time-resolved fluorescence resonance energy transfer (TR-FRET) assay and the conceptually similar bioluminescence resonance energy transfer (BRET) assay employ genetically tagged HDAC proteins for signal generation and do not depend on substrate conversion, both assays eliminate any complications that could arise from copurified HDAC impurities and thereby ensure the reliable identification of HDAC10 binders. For basic assay principles see Figure 4A,B. Further, Manfred Jung and coworkers developed the first fluorescence-based HDAC10 assay suitable for high-throughput screening.^109^ The respective assay relies on the enzymatic conversion of a specifically developed aminocoumarin-labeled acetyl-spermidine (Ac-spermidine-AMC) substrate suitable to measure the polyamine deacetylase activity of HDAC10. The basic principle of this assay is shown in Figure 4C.^109^ Notably, this assay was quickly adopted by contract research organizations who offer enzymatic profiling of HDACs and is therefore commercially available.^110^ However, in contrast to the TR-FRET HDAC10 assay (Miller lab) as well as the fluorescence-based HDAC10 assay (Jung lab) which use human recombinant HDAC10, only the BRET assay utilizes living cells that express nanoluciferase (NanoLuc)-tagged HDAC10. Thus, this assay additionally provides information concerning the cellular target engagement of the tested substances and should thereby provide more meaningful HDAC10 inhibition data in terms of isoform selectivity prediction *in vivo*.

**Figure 4:**
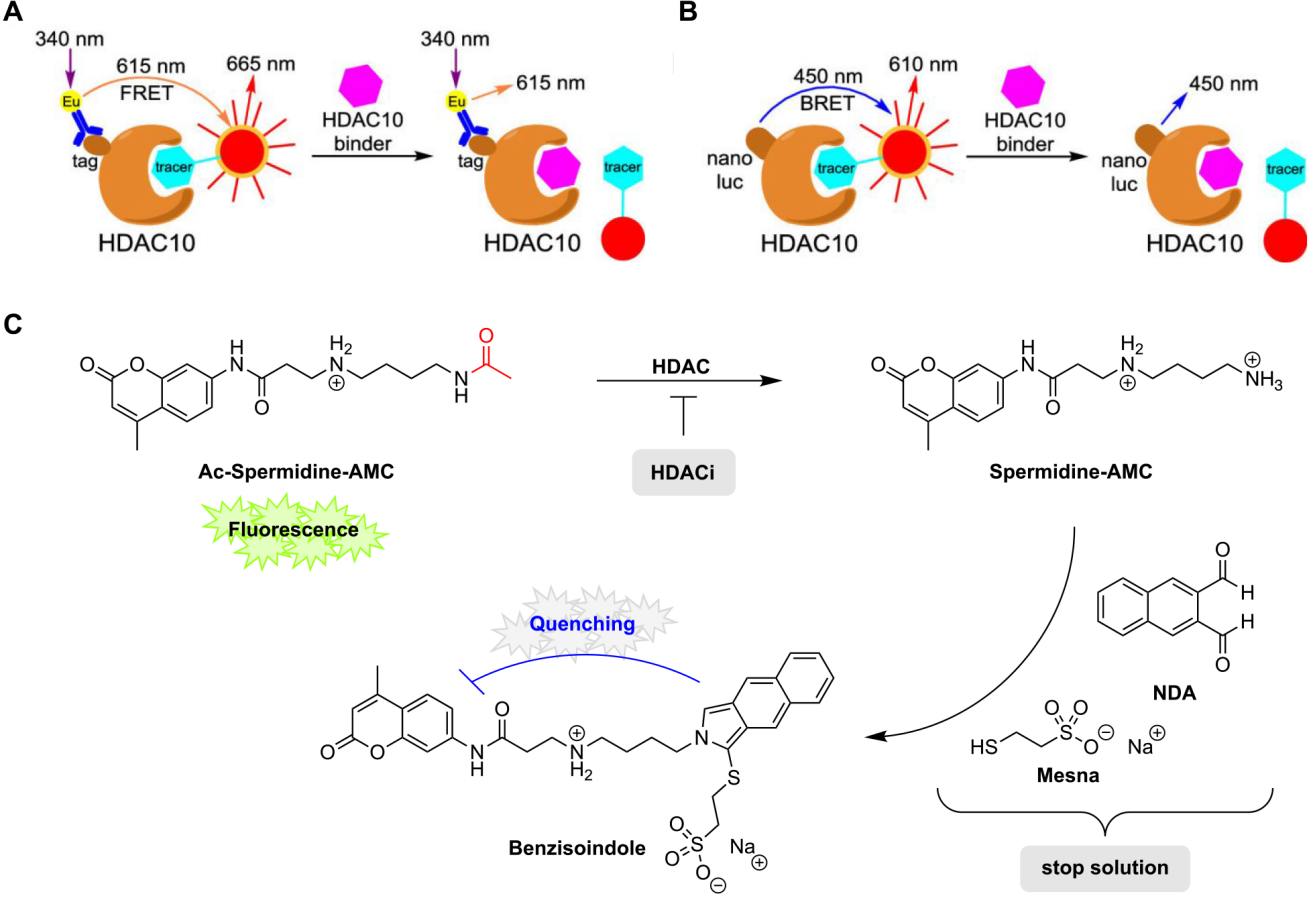
Recently developed HDAC10 assays. **A**) TR-FRET assay developed by Aubry K. Miller and coworkers.^108^ An Europium-labeled anti-GST tag antibody binds to the GST-tagged HDAC10. Upon binding of the dye-labeled tracer, irradiation (λ = 340 nm) leads to a FRET signal between the Europium (λ_em_ = 615 nm) and the dye-labeled tracer (λ_em_ = 665 nm). In the presence of a competitive HDAC10 binder, FRET is disrupted. **B**) BRET assay developed by Aubry K. Miller and coworkers.^108^ Cells expressing NanoLuc-tagged HDAC10 are treated with a dye-labeled tracer. Upon tracer binding a BRET signal is observed. In the presence of a HDAC10 binder, which competes with the tracer ligand, BRET is disrupted. **C**) Basic principle of the fluorescence-based HDAC10 assay developed by Manfred Jung and coworkers.^109^ Ac-spermidine-AMC is incubated with HDAC10 and thus deacetylated. The addition of the stop solution containing naphthalene-2,3-dialdehyde (NDA) and Mesna, leads to the conversion of spermidine-AMC to a substituted benzisoindole which quenches fluorescence intramolecularly. Fluorescence is measured at λ_ex_ = 330 nm, λ_em_ = 390 nm. Subfigures **A**) and **B**) are reprinted with permission from Géraldy, M. et al., *J. Med. Chem.* **2019**, 62(9), 4426–4443.^108^ Copyright © 2019, American Chemical Society.

### Novel Assay Formats to Investigate the Fatty-Acid Deacylase HDAC11

Based on the fatty acid deacylase activity of HDAC11, Moreno-Yruela et al. were able to identify an ε-*N*-myristoyllysine substrate that is efficiently cleaved by HDAC11 (see Figure 5A).^87^ Further, Kutil et al. reported a continuous fluorescence-based HDAC11 assay (Figure 5B) using an internally quenched peptide substrate derived from the known myristoylation site TNFα-Lys20.^111^ Additionally, Son et al. reported an HPLC quantified HDAC11 assay using a myristoyl-serine hydroxymethyl transferase 2 (SHMT2) derivative as substrate (see Figure 5C).^112^ Taken together, these assays rely on more “natural” substrates of HDAC11 and should therefore provide more valid information concerning HDAC11 inhibition than older, trifluoroacetyllysine substrate-based assays.

**Figure 5:**
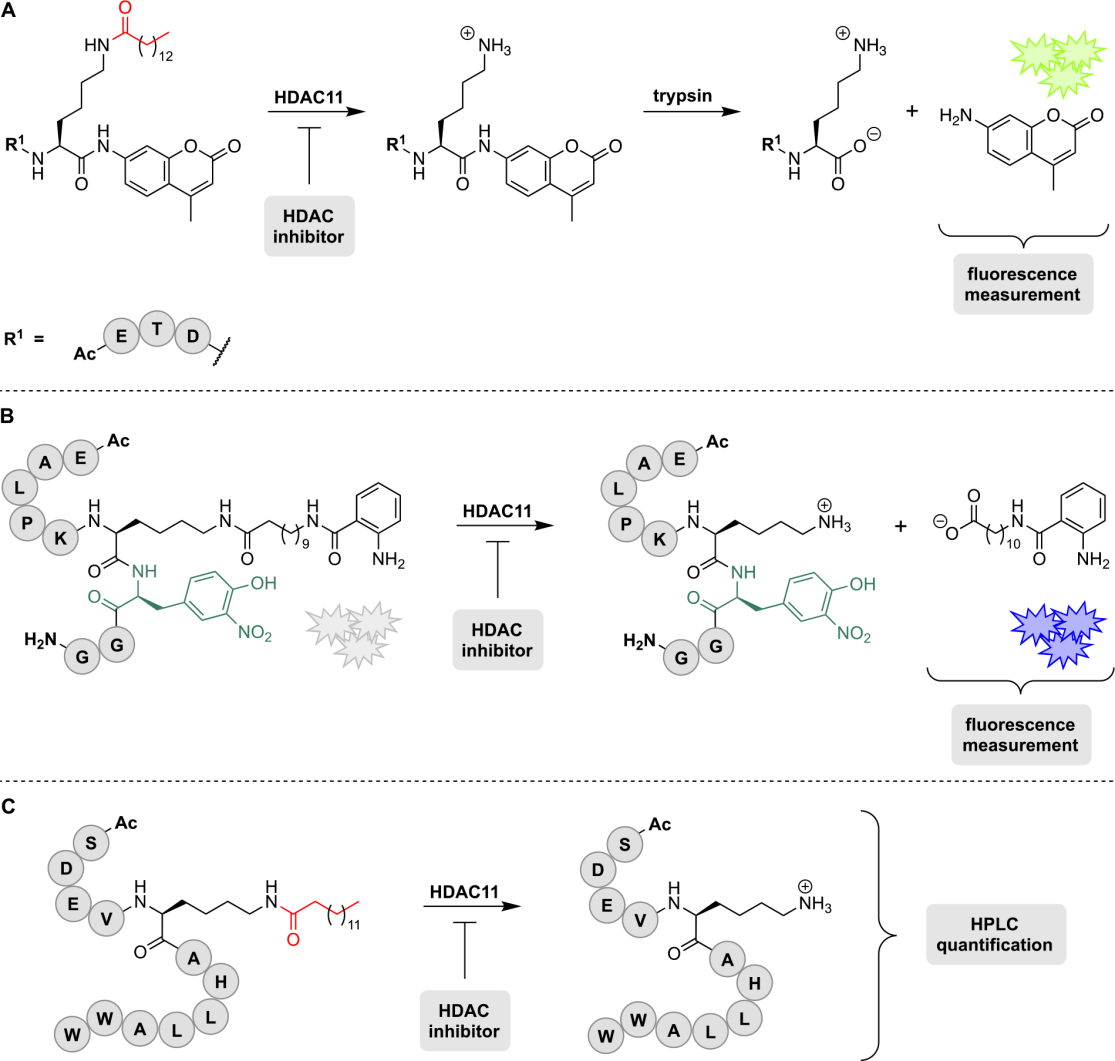
Recently developed HDAC11 assays. **A**) AMC-based fluorescence assay developed by Moreno-Yruela et al., the sequence of the dihydrolipoyl transacetylase (DLAT) derived peptide is Ac-ETDK­(myr)-AMC.^87^ Following the cleavage of the ε-*N*-myristoyllysine, the C-terminal lysine is recognized by trypsin and the fluorophore (AMC) is cleaved. The resulting fluorescence signal is measured and used to generate inhibitor concentration–response curves. **B**) Kutil et al. developed a continuous fluorescence-based assay using an internally quenched TNFα-derived peptide derivative (sequence: Ac-EALPKK­(modified)-3-nitrotyrosine-GG-NH_2_).^111^ In the native substrate, fluorescence is quenched. To this end, 3-nitrotyrosine (“Y_N_”; teal colored amino acid) was included in the peptide sequence at the +1 position of the ε-*N*-modified lysine residue. Upon incubation with HDAC11, the fluorophore (κ-*N*-anthraniloylated undecanoic acid) is cleaved resulting in an increase of fluorescence (λ_ex_ = 330 nm, λ_em_ = 430 nm). In the presence of an HDAC11 inhibitor less fluorophore is cleaved resulting in an overall reduced fluorescence signal. **C**) Son et al. reported another *in vitro* assay based on substrate conversion; the sequence of the serine hydroxymethyl transferase 2 (SHMT2) derived peptide is Ac-SDEVK­(myr)­AHLLAWW.^112^ HDAC11 is incubated with the myristoylated peptide substrate. Following incubation time, the amount of converted substrate is quantified via HPLC.

### Slow-Binding HDAC Inhibitors

Especially HDAC1–3 inhibitors often display slow-binding characteristics but there are also examples for slow-binding HDAC6 and HDAC11 inhibitors.^34,87,104,113–120^ Slow-binding inhibitors associate to and/or dissociate from their target enzymes slowly.^100^ This causes a delay in enzyme–inhibitor equilibration and essentially leads to a time dependence of enzyme inhibition data (“IC_50_ shift”).^34,100^ Since these inhibitors are slow to engage their target, their true potency is usually underestimated in standard end-point assays.^100,121^ However, the failure to properly assess such slow-binding properties can result in misleading structure–activity relationships (SAR) concerning both potency and selectivity.^100,103,122^ By now, there are several methods available to assess the slow-binding characteristics of HDACi including IC_50_ shift experiments and continuous HDAC assays.^123^ Based on the continuous monitoring of substrate conversion, the so-called *progression method* allows to investigate the binding mechanism (e.g., simple slow-binding, induced-fit etc.) as well as tight-binding properties (e.g., *Jump-Dilution* experiments) of the respective inhibitor.^34,114,115,123^ These experiments provide valuable information concerning the binding affinity of the inhibitor, its drug-target residence time and, by extension true potency and isoform selectivity.^100,116,118^ A good example for misleading SAR due to slow-binding characteristics is RGFP966 (**6**, see Figure 6C below) which is commercialized as HDAC3-selective probe.^124,125^ However, recent investigations of its binding kinetics at HDAC1–3 suggest that RGFP966 is actually a potent HDAC1–3 inhibitor and not a HDAC3-selective probe.^103^ Accordingly, the kinetic evaluation of slow-binding HDACi is necessary in order to identify isoform selective HDACi and thus validate them as chemical probes.

### Class I HDACs (HDAC1–3) - Beyond Isoform Selectivity

Besides their slow-binding characteristics, HDAC1–3 operate in the context of corepressor complexes (see above) and it has been shown that the formation of these corepressor complexes leads to conformational changes of the respective HDAC enzyme and results in the activation or increase of its deacetylase activity.^42,45,48,126^ Indeed, recent findings suggest that certain HDACi preferably bind to specific corepressor complexes or in some cases to the free enzyme which is integrated into a corepressor complex subsequently.^127–129^ Moreover, a new study of Pytel et al. found that HDACi address different corepressor complexes with different affinity despite targeting the same enzyme.^130^ Furthermore, both potency and selectivity of the investigated HDACi toward these corepressor complexes seems to be context dependent and changes, in some cases immensely, with varying concentrations of allosteric regulators such as inositol phosphates (e.g., inositol hexaphosphate). Collectively their results indicate that assessing the HDAC selectivity profile of class I HDACi in traditional *in vitro* HDAC assays might be insufficient for the characterization of potential tool compounds since each HDAC-co-repressor complex performs distinct biological functions with little redundancy.^130^ Thus, biological outcomes are most likely determined by the extent to which specific HDAC-co-repressor complexes are affected by the HDACi in question. Consequently, both binding kinetics and corepressor complex selectivity need to be carefully considered while evaluating HDAC1–3 inhibitors (see section “Recommended Tool Compounds; HDAC1–3”).

### Cellular Target Engagement of HDACi

By now, thousands of acetylation and acylation sites as well as various protein substrates of specific HDAC enzymes have been identified.^131,132^ Therefore, the selectivity profile of HDACi determined by *in vitro* HDAC assays should routinely be validated in a cellular environment using specific HDAC substrates as a readout. These include histone proteins such as acetyl histone H3 (HDAC1–3),^1,2^ which can be analyzed globally or by using specific acetylation sites such as H3K18ac, H3K27ac, or H3K36ac,^133^ acetyl p53 (HDAC1–3, HDAC8),^1,2,16^ acetyl SMC3 (HDAC8),^61^ acetyl α-tubulin (HDAC6),^2,11^ acetyl Hsp90 (HDAC6),^80^ myristoyl SHMT2 (HDAC11),^132^ and many others.^1,2,131^ HDACi usually only impact the acylation status of a specific subset of these potential substrates which is determined by the unique selectivity profile of the HDACi in question.^134^ For example, treatment with a highly selective HDAC6 inhibitor would cause elevated levels of acetyl α-tubulin but have no impact on acetyl histone H3.^135^ The quantification of these effects is—among others—possible *via* Western blot or quantitative mass spectrometry.^134,135^

### Excluded Isoforms and Modalities

As mentioned above, the in vitro characterization of both HDAC8 inhibitors and class IIa HDAC inhibitors relies on the use of highly artificial, easily scissile trifluoroacetyllysine substrates. However, since class IIa HDAC isoforms probably function as “reader” domains without deacetylase activity in vivo and HDAC8 is effectively a fatty acid deacylase, these assays are very likely unable to properly assess and predict the in vivo HDAC isoform selectivity and specificity of the respective HDACi. Therefore, we decided not to discuss or recommend tool compounds for the class IIa isoforms HDAC4, -5, -7, and -9 as well as HDAC8. Additionally, we decided not to discuss targeted protein degradation of HDACs (e.g., using proteolysis targeting chimeras and molecular glues) as well as HDAC knockout or knockdown techniques since we feel these methods are beyond the scope of this article.

## Recommended Tool Compounds

### Class I HDACs (HDAC1–3)

Generally, the design of class I selective HDACi is easily accomplished by the selection of an appropriate ZBG. Currently, the most common choice is the *ortho*-aminoanilide moiety e.g., featured by the National Medical Products Administration (NMPA)-approved HDACi tucidinostat or the late-stage clinical candidate entinostat (see Figure 6).^2^ Another emerging group of class I selective inhibitors are compounds bearing a hydrazide-based ZBG (see Figure 9A).^105,136^ Notably, isoform selectivity *within* HDAC class I often requires both the careful fine-tuning of shape, size, and polarity of the foot-pocket unit (FPU) and the kinetical evaluation of these inhibitors in order to analyze their target engagement over time.^114,116,127^ A small selection of commercially available *ortho*-aminoanilide HDACi as well as their isoform selectivity profile toward HDAC1–3 determined *via* standard HDAC assays (see above) is summarized in Figure 6. Notably, *ortho*-aminoanilide HDACi usually do not inhibit HDAC8 or HDAC10 to a relevant extent, even though particularly older literature suggests otherwise.^129,137–139^ (see section “*In Vitro* Profiling of HDAC Inhibition”).

**Figure 6:**
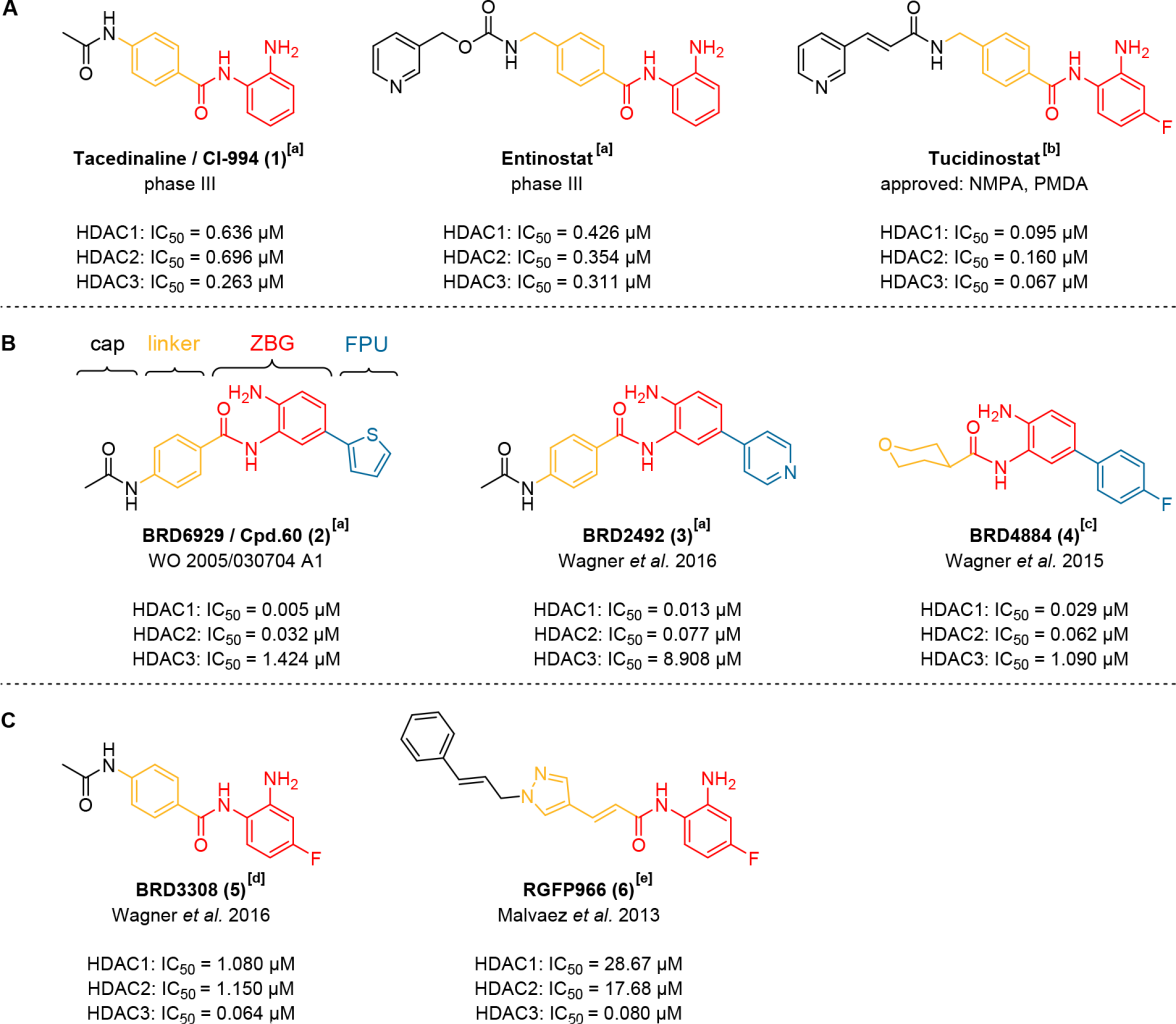
Structures of selected, commercially available *ortho*-aminoanilide HDACi with various selectivity profiles toward HDAC1–3.^2,116,125,138,146,147^ Substances are each labeled with their trade name. **A)** Well-established HDAC1–3 inhibitors. **B)** Selected *ortho*-aminoanilide HDACi bearing a “foot-pocket” unit (FPU). **C)** Selected *ortho*-aminoanilide HDACi which are commercialized as HDAC3-selective probes. IC_50_ values are taken from the following references: [a] Schäker-Hübner et al. 2022;^102^ [b] Ning et al. 2012;^146^ [c] Wagner et al. 2015;^116^ [d] Wagner et al. 2016;^138^ [e] Bowers et al. 2015.^124^ Notably, the reported isoform profile data result from different HDAC end-point assays using varying conditions as well as preincubation times and should therefore be interpreted with appropriate caution.

Tacedinaline (**1**) or CI-994 is a classic *ortho*-aminoanilide HDACi and has been intensely investigated since the early 1990s (Figure 6A).^2^ Over the years, the isoform profile of **1** has been evaluated by several research groups in *in vitro* HDAC assays.^2,9,102,116^ Collectively, their results indicate that **1** is a nonselective HDAC1–3 inhibitor. Besides that, there are two independent reports concerning the slow-binding characteristics of **1** including its drug-target residence time at HDAC1–3 and therefore kinetic selectivity: Wagner et al. simulated the target engagement profile of **1** for HDAC1–3 (see Figure 8 below) and concluded that **1** is a nonselective, tight-binding inhibitor of HDAC1–3 with prolonged target engagement on all three isoforms.^138,140^ In contrast, the data acquired by Moreno-Yruela et al. suggest a kinetic preference for HDAC1 over HDAC2 and HDAC3 due to the comparably long dissociative half-life (Diss. *t*_1/2_)^141^ of **1** at HDAC1.^114^ Furthermore, chemoproteomics studies revealed that **1** preferably binds the corepressor complexes CoREST and NCoR in a time-dependent manner but seems not to engage the Sin3A complex.^137^ Further, a recent study by Pytel et al. found the same trend. However, the study also shows that the corepressor complex selectivity profile of **1** is dependent on the allosteric regulator inositol phosphate. This indicates that there might be profound differences between its observed selectivity profile *in vitro* and its actual selectivity profile *in vivo*.^130^ Besides these kinetic studies, **1** has been thoroughly profiled e.g., concerning its physicochemical properties,^102^ pharmacokinetic characteristics,^142,143^ and *in vitro*/*in vivo* toxicity both in preclinical (animal) models^138,143,144^ and in humans.^145^ Furthermore, **1** was under clinical investigation in the early 2000s for the treatment of different kinds of cancer (e.g., NCT00005624 and NCT00004861 (phase II); NCT00005093 (phase III)). Overall, **1** is a very well characterized, albeit not especially potent, HDAC1–3 inhibitor and therefore a suitable tool compound for the combined investigation of these class I HDAC isoforms. However, given the preference of **1** for certain corepressor complexes, biological results should be interpreted with care.

Entinostat (Figure 6A) is a late stage clinical candidate HDAC1–3 inhibitor which was first reported in the late 1990s.^2,148^ Accordingly, its isoform profile has been evaluated by several research groups in *in vitro* HDAC assays, consistently concluding that entinostat is a nonselective HDAC1–3 inhibitor.^2,9,102,103,146,149^ Additionally, entinostat has been investigated concerning its slow-binding characteristics toward HDAC1–3, overall confirming the isoform selectivity profile obtained in standard end-point HDAC assays.^103,129,140,149^ Furthermore, chemoproteomics studies have shown that entinostat, similar to tacedinaline, preferably binds the corepressor complexes CoREST and NCoR in a time-dependent manner but seems not to engage the Sin3A complex.^137^ However, similar to **1**, the corepressor complex selectivity profile of entinostat is dependent on inositol phosphate suggesting relevant differences between its *in vitro* and *in vivo* selectivity profile.^130^ There is also a comprehensive data set available concerning the pharmacokinetic characteristics^150–152^ and the *in vivo* toxicity^151,152^ of entinostat. Besides that, entinostat is under intense evaluation in clinical studies (ClinicalTrials.gov currently lists more than 70 clinical trials involving entinostat^153^) for the treatment of various kinds of cancer (e.g., NCT03250273 and NCT03838042 (phase II); NCT02115282 (phase III)). Taken together, entinostat is a very well characterized, moderately potent HDAC1–3 inhibitor and therefore a suitable tool compound for the combined investigation of these class I HDAC isoforms. However, considering its preference for certain corepressor complexes, biological results should be interpreted with caution.

In contrast to tacedinaline and entinostat, tucidinostat (chidamide, Epidaza) is an approved drug: Tucidinostat (Figure 6A) is approved in China for the treatment of relapsed or refractory peripheral T-cell lymphoma (PTCL) and the treatment of hormone-receptor-positive (HR+) breast cancer in combination with exemestane.^154–157^ Additionally, tucidinostat is approved in Japan for the treatment of relapsed or refractory adult T-cell leukemia-lymphoma.^158^ Furthermore, tucidinostat is under intense clinical investigation (ClinicalTrials.gov currently lists more than 200 clinical trials involving tucidinostat^159^) among others for the treatment of diffuse large B-cell lymphoma (e.g., NCT04231448 (phase III)) in combination with several other cytotoxic drugs.^160^ According to its isoform profile, tucidinostat is a potent, nonselective HDAC1–3 inhibitor.^140,146^ Given its status as an approved drug, tucidinostat has been thoroughly profiled e.g., concerning its pharmacological effects on various cellular proteins, cytokines and receptors, pharmacokinetic characteristics,^161–164^ and *in vivo* toxicity in humans.^161,165^ However, in comparison to the detailed information regarding the pharmacokinetic profile and *in vivo* efficacy and toxicity of tucidinostat, there is hardly any information concerning the binding kinetics of tucidinostat at HDAC1–3 and/or the different corepressor complexes. To the best of our knowledge there is no data available concerning the kinetic HDAC1–3-selectivity of tucidinostat but there are two reports available concerning the binding of tucidinostat to the corepressor complexes CoREST, NuRD, Sin3A, MiDAC, or NCoR: In 2022, Lechner et al. investigated off-targets of common HDACi. In this context, they also evaluated the binding of tucidinostat to certain corepressor complexes in comparison to the binding of tucidinostat to isolated HDAC1 and HDAC3. Based on the presented data, tucidinostat binds to HDAC1–3 with similar potency. Further, tucidinostat appears to bind with lower affinity to the MiDAC, NuRD and Sin3A corepressor complexes than to HDAC1 alone, while it shows comparable affinity to the NCoR corepressor complex as to the isolated HDAC3 enzyme. Notably, Tango Therapeutics Inc. (WO 2024/030659 A1)^166^ investigated tucidinostat in comparison to their CoREST-selective HDACi TNG260 (see below) and came to similar conclusions. However, although this could indicate a certain downstream selectivity *in vivo*, neither Lechner et al. nor the authors of patent WO 2024/030659 A1 discussed these results in detail.^139,166^ Given the collective data and its status as an approved drug, tucidinostat seems to be suitable to investigate tumor metabolomics and antineoplastic (combination)­therapies *in vivo*, especially since isotopically labeled reference standards of tucidinostat (tucidinostat-*d*_4_, tucidinostat-^13^C­(6))^167,168^ are commercially available. Besides that, tucidinostat is less suitable for basic biological studies as well as target validation due to the lack of comprehensive data concerning its binding kinetics and corepressor complex selectivity.

Compounds like BRD6929 (**2**), BRD2492 (**3**), and BRD4884 (**4**) (Figure 6B) target the so-called “foot-pocket” and are commercialized as HDAC1/2 selective HDACi. From a structural point of view, the apparent selectivity profile of these compounds is achieved by introducing a so-called “foot-pocket” unit (FPU; e.g., the 2-thienyl-group of cpd. **2**) to the *ortho*-aminoanilide ZBG (see Figures 6B and 7B).^1,2,49,50,147,169^

**Figure 7:**
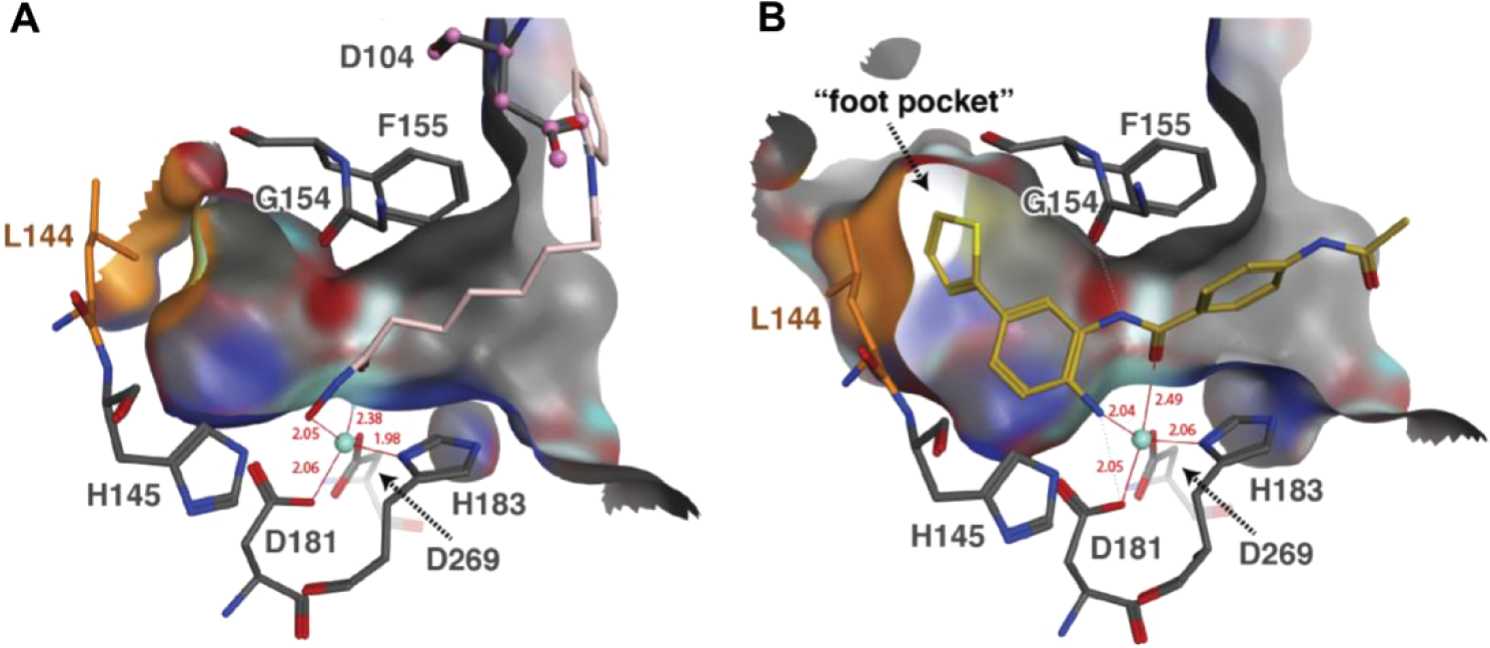
**A**) Structural analysis of the pan-HDACi vorinostat bound to HDAC2 (PDB: 4LXZ). **B**) Structural analysis of the *ortho*-aminoanilide HDACi BRD6929 (**2**) bound to HDAC2 (PDB: 4LY1). The binding pocket surface of HDAC2 is shown in gray, the catalytic Zn^2+^-ion is shown as a turquoise sphere, key HDAC2 pocket residues are shown as gray sticks, and ligands are colored pink (vorinostat) or gold (**2**), respectively. In the case of **2**, leucine 144 (L144; shown in orange) is able to change its orientation and thereby opens up the “foot pocket”. Reproduced with permission from Lauffer, B. E. L. et al., *J. Biol. Chem.* **2013**, 288 (37), 26926–26943.^149^ Copyright © 2013 ASBMB. Currently published by Elsevier Inc.; originally published by American Society for Biochemistry and Molecular Biology.

BRD6929 (**2**) was first described in 2005 by MethylGene, Inc. (WO 2005/030704 A1)^147^ and is usually considered as a potent, slow-binding, HDAC1/2 selective HDACi.^49,116,118,149^ Beyond that, there are two independent reports concerning the slow-binding characteristics of **2** including its drug-target residence time at HDAC1/2: According to the results of Wagner et al., **2** shows remarkably long target engagement on HDAC1 (drug-target residence time (*T*_1/2_) > 2400 min) and even more so on HDAC2 (*T*_1/2_ ∼ 4800 min). However, and in contrast to BRD4884 (see below) the authors did not report a kinetic selectivity for either HDAC isoform.^116,118^ Additionally, Lauffer et al. report comparably long drug-target residence times on HDAC1 (*T*_1/2_ = 1207 min) and HDAC2 (*T*_1/2_ = 1259 min) and conclude, that **2** is a slow tight-binding HDAC1/2 inhibitor which exhibits sustained HDAC1/2 inhibition greater than 20 h.^149^ Additionally, basic pharmacokinetic data are available for **2** including the area under the plasma concentration curve (AUC) and the maximum plasma concentration (*C*_max_) after peroral (rat) and intraperitoneal (i.p.) application (mice), the plasma half-life (*t*_1/2_) of **2** in rats and mice, as well as its clearance (CL), bioavailability (*F*), and volume of distribution (Vd_ss_) in rats.^49,170^ Further, **2** seems to be able to cross the blood-brain-barrier (BBB) in mice^170^ and is well tolerated up to a dose of 30 mg/kg (i.p.) in rats and 45 mg/kg (i.p.) in mice.^49,170^ Furthermore, **2** showed no activity at CYP enzymes or the *h*ERG potassium ion channel.^49^

However, a recent study showed that the alleged HDAC1/2 selectivity of **2** might very well be an assay-artifact.^127^ As mentioned above, HDAC assays usually utilize isolated HDAC enzymes. In the case of HDAC3, however, assays are performed using the HDAC3:NCoR2 protein complex due to the low deacetylase activity of isolated HDAC3.^2,45,48,98,102,103,127^ Interestingly, the study of Payne and Mazitschek demonstrated that compound **2** binds with similarly high affinity to free HDAC1, HDAC2, and HDAC3.^127^ Furthermore, the authors report that HDACi-bound HDAC3 associated faster with NCoR2 than free HDAC3 and that **2** remained stably bound to the HDAC3:NCoR2 corepressor complex.^127^ As a result, Payne and Mazitschek conclude that **2** can only adopt its high-affinity binding pose if the backbone of HDAC3 is sufficiently flexible to allow the ligand to slip into position. This is probably only possible in the free state of the enzyme, since binding to a complex partner (e.g., NCoR2) rigidifies the protein. Consistently, the authors provide evidence that **2** only weakly binds to HDAC1 and HDAC2 when preassembled in the CoREST complex and conclude that the biological effects of **2** might predominantly result from the *de novo* assembly of inhibited corepressor complexes.^127^ A complementary study of Pytel et al. even suggests that the isoform-selectivity pattern of **2** might be flipped toward HDAC3 *in vivo*: According to their data, **2** does not significantly engage the most relevant HDAC1/2 corepressor complexes (e.g., CoREST, MiDAC, and NuRD) under *in vivo* conditions while still engaging the HDAC3:NCoR complex with a low micromolar IC_50_ value.^130^ Thus, albeit very well characterized *in vitro* and *in vivo*, **2** cannot be recommended as tool compound to elucidate the specific biological roles of HDAC1/2 in contrast to HDAC3.

BRD2492 (**3**), first described by Wagner et al. in 2016, is a potent, slow-binding inhibitor of HDAC1/2 with a favorable physicochemical profile—especially compared to **2**.^102,138^ Further, investigations concerning the binding kinetics of **3** revealed a two phase kinetic selectivity profile: In a first phase (*t* = 0–10 h), **3** shows kinetic selectivity for HDAC1 over HDAC2/3. However, this phase is followed by a much longer second phase (*t* > 10 h) of increasingly good kinetic selectivity for HDAC2 over HDAC1/3 caused by the “slower-on/slower-off” kinetic at HDAC2 (*T*_1/2_ ∼ 30 h vs ∼ 7 h (HDAC1)). The simulated target engagement profile of **3** compared to **1** (see above) and **5** (see below) is depicted in Figure 8.^138^ The remarkably long target engagement time of **3** is especially interesting, since its plasma half-life after i.p. application (mice) is comparably short (*t*_1/2_ = 1.59 h).^138^ Notably, HDAC1–3 knockdown experiments using small-interfering RNA (siRNA) suggest that the biological effects of **3** on rat INS-1E insulinoma cells (treatment time 48 or 72 h) are most likely not related to HDAC3 inhibition.^138^ These results substantiate the HDAC1/2 selectivity of **3** in a cellular context, which was previously only evident from biochemical HDAC assays. Additionally, Wagner et al. report a few other basic pharmacokinetic parameters for **3** including plasma protein binding, plasma stability, and liver microsome stability (human, mice, rat).^138^ Overall, **3** is a moderately well characterized HDACi that might be a suitable tool compound for HDAC1/2 after further characterization of its corepressor complex selectivity profile (see compound **2**). Until then, biological outcomes should be interpreted with caution and verified by complementary approaches.

**Figure 8:**
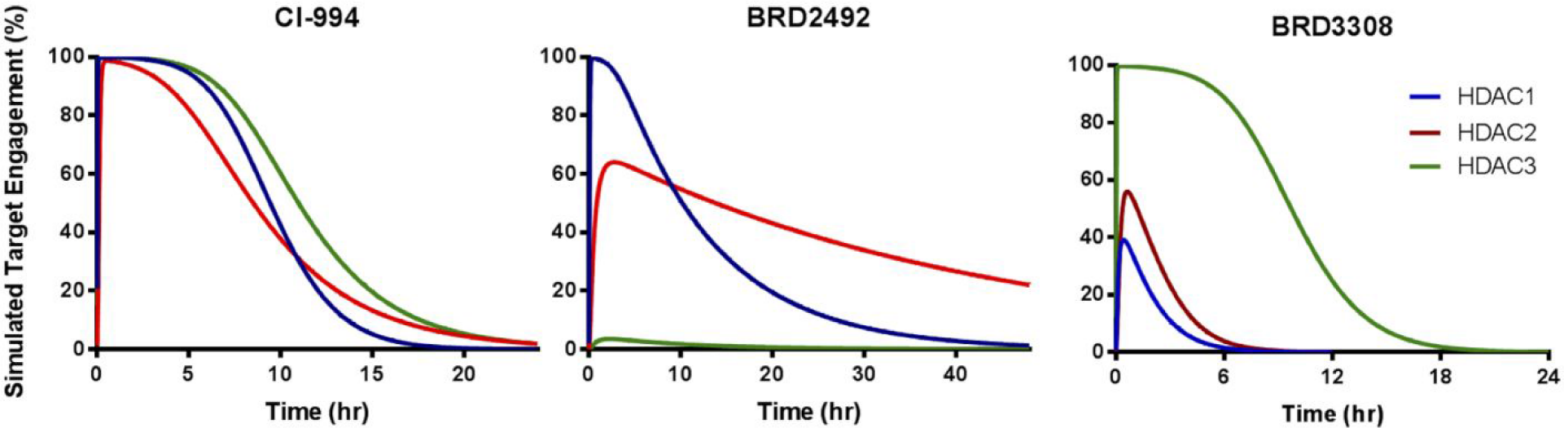
Simulated target engagement profiles (HDAC1–3; plasma) for **1** (CI-994; left), **3** (BRD2492, middle), and **5** (BRD3308, right) for a dose of 10 mg/kg (i.p.) in mice. Reprinted with permission from Wagner, F. F. and Lundh, M. et al., *ACS Chem. Biol.* **2016**, 11 (2), 363–374.^138^ Copyright © 2016, American Chemical Society.

Similar to compound **2** and **3**, BRD4884 (**4**) is a potent, slow-binding inhibitor of HDAC1/2. Compound **4** was first described by Wagner et al. in 2015 as a brain penetrant, kinetically selective HDAC2 inhibitor.^116^ Additionally, in their follow-up paper concerning the structure–activity relationships of related HDAC1/2 inhibitors, Wagner et al. report a crystal structure of **4** bound to human HDAC2 (PDB: 5IWG).^118^ Further, their kinetic evaluation of **4** revealed a three phase kinetic selectivity profile: An initial phase (*t* = 0–1 h) of good kinetic selectivity for HDAC1 over HDAC2/3 is followed by a crossover stage with mixed target engagement at HDAC1 and HDAC2 and a third phase (*t* > 3 h) of high and sustained kinetic selectivity for HDAC2 over HDAC1/3.^116,118^ This third phase mainly originates from the “slower-on/slower-off” kinetic at HDAC2 (*T*_1/2_ = 143 min vs 20 min (HDAC1)), an effect which was previously described for compound **3**.^116,118,138^ In addition to these kinetic studies, the authors provide the basic pharmacokinetic parameters AUC, *t*_1/2_ and *C*_max_ in mice plasma and brain, respectively.^116^ Besides that, **4** is well tolerated in a daily dose of 10 mg/kg (mice; i.p. or peroral (p.o.)),^116,171,172^ shows low potential for cardiac toxicity (*h*ERG) and high target specificity versus a panel of potential off-targets.^116^ In summary, **4** is a moderately well characterized, brain penetrant HDAC1/2 inhibitor that could be a suitable tool compound after further characterization. However, given the complex kinetic isoform selectivity profile of **4** and the present lack of data concerning its corepressor selectivity (see compound **2**), biological outcomes should be interpreted with caution.

Compounds BRD3308 (**5**) and RGFP966 (**6**) (Figure 6C) are commercialized as HDAC3-selective probes. Interestingly, some authors even argue that the addition of a fluorine to the *ortho*-aminoanilide ZBG promotes HDAC3 selectivity.^2,52,124,125^ However, considering the structure of tucidinostat (Figure 6A) and the recent evaluations of Moreno-Yruela et al. (see section “RGFP966 (**6**)” below) this SAR concept should be reevaluated.^103^

Compound BRD3308 (**5**) was first introduced in a patent in 2014 and investigated in the following years by different research groups concerning its potential use to treat diabetes.^54,138,140^ **5** is a slow-binding HDAC1–3 inhibitor whose kinetic HDAC3 selectivity largely results from its “faster-on/slower-off” kinetics at HDAC3 compared to HDAC1/2 (HDAC3: *T*_1/2_ = 79 min vs HDAC1: *T*_1/2_ ∼ 2.5 min and HDAC2: *T*_1/2_ = 13 min).^138,140^ The simulated target engagement profile of **5** compared to **1** and **3** (see above) is depicted in Figure 8.^138^ This observed selectivity for HDAC3 is even more pronounced when lower doses of **5** are applied (see Wagner et al. 2016; Supporting Figure S6) and has been verified *in vitro* by siRNA-mediated knockdown experiments.^138^ Besides these kinetic studies, there are also basic information available concerning the pharmacokinetics and pharmacodynamics of **5** including: AUC, *t*_1/2_, and *C*_max_ in mice plasma and brain, respectively;^138,140^ plasma protein binding, plasma stability, and liver microsome stability (human, mice, rat).^138^ Furthermore, **5** is well tolerated in mice in daily doses up to 10 mg/kg for 2 weeks (i.p.) followed by 20 weeks of treatment twice a week (i.p.).^173^ In summary, **5** is sufficiently well characterized, brain penetrant, shows convincing kinetic selectivity for HDAC3 *in vitro* and has a favorable toxicity profile in mice. Given these properties, **5** might be a suitable tool compound for the investigation of HDAC3. However, due to the lack of data concerning the corepressor selectivity of compound **5**, biological outcomes need to be verified by complementary approaches.

RGFP966 (**6**), which is marketed as an HDAC3-selective probe, was first introduced by Malvaez et al. as an HDAC3-selective inhibitor in 2013, without fully disclosing the selectivity profile of the compound.^125^ Two years later, the HDAC isoform profile of **6** was reported by Bowers et al.^124^ However, by then **6** was already under intense scrutiny as a “HDAC3-selective” inhibitor e.g., in several animal studies.^174–177^ Although some pharmacokinetic and pharmacodynamic data are available for **6**, the overall data set is surprisingly limited: **6** is a brain penetrant HDACi (brain to plasma ratio: 0.43) which reaches *C*_max_ in the brain of mice (10 mg/kg; subcutaneously (s.c.)) 15 to 30 min after application (*t*_1/2_ ∼ 45 min).^124,125^ Further, **6** seems to be well tolerated in daily doses of 20 mg/kg (p.o.),^178^ 30 mg/kg (i.p.),^176^ as well as 25 mg/kg (s.c.; 3x/week)^177^ in mice. However, Moreno-Yruela et al. recently investigated **6** concerning its potency and isoform selectivity by comparing the results from standard end-point assays and continuous HDAC assays. Their findings suggest that **6** is actually a potent HDAC1–3 inhibitor and not a HDAC3-selective probe.^103^ This is especially problematic since the effects of **6** on learning and behavior are usually attributed to the selective inhibition of HDAC3.^125,179,180^ Consequently, the use of **6** as a HDAC3-selective tool compound cannot be recommended until the compound is sufficiently well characterized concerning its (kinetic) HDAC selectivity profile as well as corepressor selectivity.

Besides the well-established *ortho*-aminoanilides, alkyl-hydrazides are an emerging group of remarkably potent HDACi. Interestingly, the length of the alkyl chain seems to determine the isoform selectivity profile of the inhibitor: While HDACi bearing a propyl hydrazide ZBG show a preference for HDAC3,^181,182^ hexyl hydrazides show increased HDAC8 inhibition,^185,186^ and further elongation of the alkyl moiety results in potent HDAC11 inhibition (see section “HDAC11” below).^112^ Selected propyl hydrazide HDACi^181,182^ as well as the HDAC3 selective 2-methylthiobenzamide HDACi reported by Liu et al.^183^ are shown in Figure 9A.

**Figure 9:**
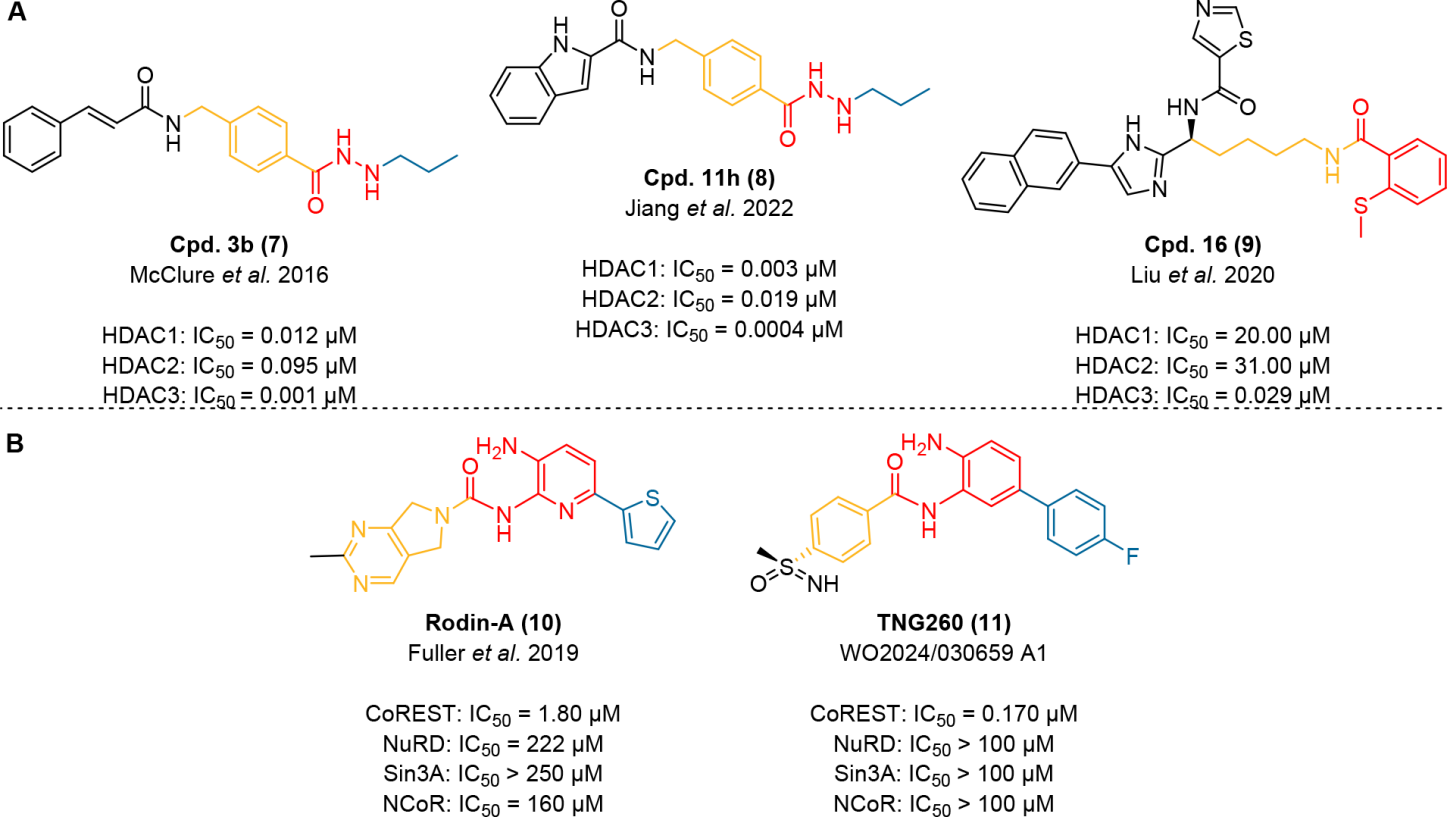
Structures of other class I HDAC addressing inhibitors. **A)** Selected HDAC3 preferential inhibitors bearing a non *ortho*-aminoanilide ZBG. Isoform profile data are taken from the respective reference listed beneath the structure.^181–183^ Notably, isoform profile data result from different HDAC end-point assays using varying conditions as well as preincubation times and should therefore be interpreted with appropriate caution. **B)** Structures of CoREST-selective HDACi. Co-repressor selectivity data are taken from the respective reference listed beneath the structure.^166,184^

The potent propyl hydrazide Cpd. 3b (**7**) was first introduced by McClure et al. in 2016 and marks the starting point for a series of publications by C. James Chou and coworkers dealing with the development of hydrazide-based HDACi including Cpd. 11h (**8**).^105,115,181,182^ In this first publication, C. James Chou and coworkers investigate the binding mode of the class I HDACi **7** at HDAC1 and HDAC3 by conducting *in vitro* *V*_max_ studies. The resulting Lineweaver–Burke plots suggest a mixed binding at HDAC1 and HDAC3. Therefore, the authors speculate that hydrazide-based HDACi additionally engage class I HDACs via an allosteric binding site unrelated to the catalytic side of these enzymes.^181^ Further, the authors show that **7** does not engage HDAC6 and provide evidence for its metabolic stability *in vitro*. Moreno-Yruela and Olsen also investigated the binding mode of **7**. They conclude that **7** is a slow-binding HDAC1–3 inhibitor. Interestingly, they observed major differences between HDAC1/2 and HDAC3 (IC_50_-shift, binding mechanism, tight-binding properties etc.) which warrant further investigation.^187^ Thus, given the overall sparse and incomplete data set, **7** is not suitable as a chemical probe. In a 2022 follow-up publication, Jiang et al. introduced another propyl hydrazide HDACi: Cpd. 11h (**8**) is a remarkably potent, slow-binding class I selective HDACi with a preference for HDAC3. The authors also explored the binding mode of **8** with similar conclusions like for **7**. Further, Jiang et al. performed an *in vivo* efficacy study and report basic pharmacokinetic and pharmacodynamic parameters including oral bioavailability (*F* ∼ 100%), AUC_0‑inf_, *C*_max_, plasma half-life (*t*_1/2_), and tissue distribution in mouse. Further, no significant body weight loss or organ toxicity was observed up to 4 mg/kg/d (p.o., 24 d, mouse). Given these promising data, **8** might be a good tool compound for class I HDACs after further characterization of its kinetic isoform selectivity and corepressor selectivity profile. However, at this point biological outcomes should be interpreted with caution.

In 2020, Liu et al. reported Cpd. 16 (**9**) as a HDAC3 selective inhibitor (see Figure 9A) with an unique 2-methylthiobenzamide ZBG.^183^ Its remarkable HDAC3 selectivity is rationalized by a co-crystal-structure of **9** bound to modified human HDAC2, engineered to mimic the active site of HDAC3 (E204_F205insY; PDB ID: 7KBH).^183^ Interestingly, this co-crystal-structure revealed an unusual monodentate Zn^2+^ coordination geometry. Additionally, the 2-methylthiobenzamide ZBG seems to induce an “in-to-out flip” of the conserved catalytic site tyrosine (Y305) which significantly expands the volume of the inhibitor binding pocket. Liu et al. conclude that the combined energetic cost of the monodentate Zn^2+^ coordination and the conformational rearrangement of the HDAC2 binding pocket causes the low activity of **9** at HDAC1 and HDAC2 and thus its observed selectivity for HDAC3.^183^ Unfortunately, **9** is also a potent inhibitor of the *h*ERG potassium ion channel (IC_50_ = 16 nM) and the authors were unable to determine basic pharmacokinetic parameters probably due to rapid metabolization.^183^ Thus, **9** has very limited utility as a tool compound for HDAC3, at least for *in vivo* studies.

In summary, selecting the right tool compound for the class I enzymes HDAC1, HDAC2, and HDAC3 remains challenging (see Table 2). Currently, there are no inhibitors available which selectively address only one of the three isoforms. Instead, most HDAC1–3 inhibitors are slow-binding HDACi that show complicated binding kinetics among these enzymes. Nevertheless, some class I HDACi exhibit kinetic selectivity for a specific class I HDAC isoform *in vitro*, implicating these substances as potential tool compounds for the respective HDAC enzyme. Furthermore, recent studies also suggest that class I HDACi are additionally characterized by their unique corepressor complex selectivity profile. These corepressor complex selectivity profiles are most likely context-dependent but are still poorly understood. Thus, when investigating class I HDACs, the most suitable tool compound needs to be selected very carefully depending on the specific scientific question, and the results obtained should be critically evaluated.

**Table 2:** Formal Information and General Assessment of Potential HDAC Tool Compounds^a^

**Section**	**Nr.**	**Original Designation or Commercial Name**	**CAS-Number**	**Relevant HDAC Isoform(s)**	**Compound Limitations**	**Recommended Tool Compound**
Class I HDACs (HDAC1–3)	**1**	Tacedinaline/CI-994	112522-64-2	HDAC1–3	- binding kinetics at HDAC1–3: inconsistent data	**(+)**
					- context-dependent co-repressor complex selectivity profile	
		Entinostat	209783-80-2	HDAC1–3	- context-dependent co-repressor complex selectivity profile	**(+)**
		Tucidinostat	1616493-44-7	HDAC1–3	- binding kinetics at HDAC1–3: largely unknown	**(+)**
					- co-repressor complex selectivity profile: sparse data set	
	**2**	BRD6929/Cpd.60	849234-64-6	HDAC1/2	- not recommended	**—**
	**3**	BRD2492	1821669-43-5	HDAC1/2	- co-repressor complex selectivity profile: unknown	**(+)**
	**4**	BRD4884	1404559-91-6	HDAC1/2	- complex, time dependent kinetic isoform selectivity profile *in vitro*	**(+)**
					- co-repressor complex selectivity profile: unknown	
	**5**	BRD3308	1550053-02-5	HDAC3	- co-repressor complex selectivity profile: unknown	**(+)**
	**6**	RGFP966	1357389-11-7	HDAC3	- not recommended	**—**
	**7**	Cpd. 3b	2044701-99-5	HDAC1–3	- not recommended	**—**
	**8**	Cpd. 11h	2763368-89-2	HDAC1–3	- binding kinetics at HDAC1–3: unknown	**(+)**
					- co-repressor complex selectivity profile: unknown	
	**9**	Cpd. 16	2498780-79-1	HDAC3	- not recommended	**—**
CoREST-selective HDACi	**10**	Rodin-A	2222756-46-7	HDAC1/2:CoREST		**+**
	**11**	TNG260	2935964-98-8			**++**
Class IIb HDACs (HDAC6)		Tubastatin A	1252003-15-8	HDAC6	- not recommended	**—**
		Nexturastat A	1403783-31-2		- not recommended	**—**
	**12**	ACY-1083	1708113-43-2		- not recommended	**—**
Class IIb HDACs (HDAC6)	**13**	T-518	2276680-91-0	HDAC6		**+**
	**14**	SE-7552	2243575-79-1			**+**
	**15**	Cpd. 6/BK-1	2444302-23-0			**+**
	**16**	Cpd. 8	2071224-39-8			**+**
	**17**	Cpd. 9	2991427-09-7			**++**
	**18**	ITF5924	2760854-72-4			**+**
Class IIb HDACs (HDAC10)	**19**	Cpd. 28	2058255-30-2	HDAC10	- not recommended	**—**
	**20**	DKFZ-711	2489335-50-2		- overall sparse data set compared to DKFZ-748 (**22**)	**(+)**
	**21**	DKFZ-728	2489335-73-9		- overall sparse data set compared to DKFZ-748 (**22**)	**(+)**
	**22**	DKFZ-748	2490709-68-5			**++**
Class IV HDAC (HDAC11)	**23**	SIS17	2374313-54-7	HDAC11	- no data available concerning (off-target) toxicity or pharmacokinetic/pharmacodynamic properties	**(+)**
	**24**	TD034	3030718-42-1		- no data available concerning (off-target) toxicity or pharmacokinetic/pharmacodynamic properties	**(+)**
	**25**	PB94	3032970-74-1		- not recommended	**—**
	**26**	14-N^C6^OH	-		- no data available concerning (off-target) toxicity or pharmacokinetic/pharmacodynamic properties	**(+)**
	**27**	Cpd. A9	2989934-63-4		-off-target toxicity: no data	**(+)**
					- unknown (oral)/mediocre (i.p.) bioavailability	

a — Not suitable as a tool compound; (+) suitable as a tool compound under certain circumstances; + suitable tool compound; ++ recommended tool compound.

### CoREST-Selective HDACi

In contrast to the HDACi depicted in Figures 6 and 9A, Rodin-A (**10**, Figure 9B) and TNG260 (**11**, Figure 9B) selectively target CoREST, a chromatin-modifying corepressor complex that contains two catalytically active HDAC1/2 enzymes.^126^ In 2019, Fuller et al. reported a series of foot-pocket targeting *ortho*-aminoanilide derivatives including RodinA (**10**) which selectively inhibits the activity of HDAC1/2 within the CoREST complex and showed strong prosynaptic effects in wild-type (WT) mice.^184^ Further, **10** was investigated regarding its effects on human erythroid and myeloid progenitors in order to predict its hematological toxicity profile in humans. Notably, **10** showed only mild hematological effects while CI-994 (**1**) and Cpd. 60 (**2**) had severe toxic effects on both myeloid and erythroid progenitor cells. Additionally, the authors provide key pharmacokinetic parameters (e.g., AUC, *C*_max_(plasma/brain), brain/plasma ratio; p.o., mouse) as well as the no observed adverse effect level (NOAEL) in Sprague–Dawley rats (45 mg/kg; 14 d) and beagle dogs (120 mg/kg; 14 d).^184^

The “CoreDAC” inhibitor TNG260 (**11**) was reported in 2022 by Tango Therapeutics. According to the related conference abstract, **11** shows 500-fold selectivity for the CoREST complex over other HDAC1-containing complexes such as NuRD and Sin3A and reverses the immune evasion phenotype in STK11 mutant nonsmall cell lung cancer (NSCLC).^128^ Like **10**, **11** was investigated regarding its effects on human erythroid and myeloid progenitors where it showed considerably less toxicity then entinostat and tucidinostat.^128,166^ Further, the 2024 patent provides key pharmacokinetic parameters of **11** in Sprague–Dawley rats, beagle dogs, and cynomolgus monkeys.^166^ Overall, **11** shows high oral bioavailability as well as low *in vivo* clearance resulting in druglike plasma half-lives (*t*_1/2_ = 4.8–8.4 h; p.o., 3 mg/kg). Also, **11** showed no relevant activity at CYP enzymes or the *h*ERG potassium ion channel (IC_50_ = 71.1 μM).^166^

Taken together, both **10** and **11** are CoREST-selective HDACi which are well characterized concerning their pharmacokinetic profiles as well as toxicity. Therefore, both compounds are suitable as tool compounds. However, given its superior CoREST selectivity, **11** might be the better choice in most cases (see Table 2).

### Class IIb HDACs – HDAC6

Although putatively “selective” HDAC6 inhibitors like tubacin^188^ have been known for over two decades, structure-based design efforts gained momentum with the development of tubastatin A in 2010. In the absence of cocrystal structures, Kozikowski and coworkers employed homology modeling of HDAC1 and HDAC6, thereby identifying a significantly wider channel rim in HDAC6 compared to HDAC1 (17.5 Å vs 12.5 Å).^189^ Leveraging this insight, they designed a series of hydroxamic acid–based HDAC6 inhibitors featuring carbazole-derived cap groups to exploit the broader channel rim and enhance selectivity over class I isoforms. The lead compound, tubastatin A (see Figure 10), incorporated a benzyl linker and a bulky tetrahydro-γ-carboline cap group. It exhibited potent and selective HDAC6 inhibition, with HDAC8 being the only other Zn^2+^-dependent isoform substantially affected. At that time, the following HDAC isoform data were reported: HDAC6 IC_50_ = 0.015 μM; HDAC8 IC_50_ = 0.854 μM; HDAC1 IC_50_ = 16.4 μM; all other HDAC isoforms IC_50_ > 30 μM. In contrast, Figure 10 shows HDAC isoform data resulting from contemporary HDAC assays (see section “*In Vitro* Profiling of HDAC Inhibition”). Notably, tubastatin A showed potential for treating neurodegenerative diseases by providing dose-dependent protection against glutathione depletion-induced oxidative stress in primary cortical neuron cultures.

**Figure 10:**
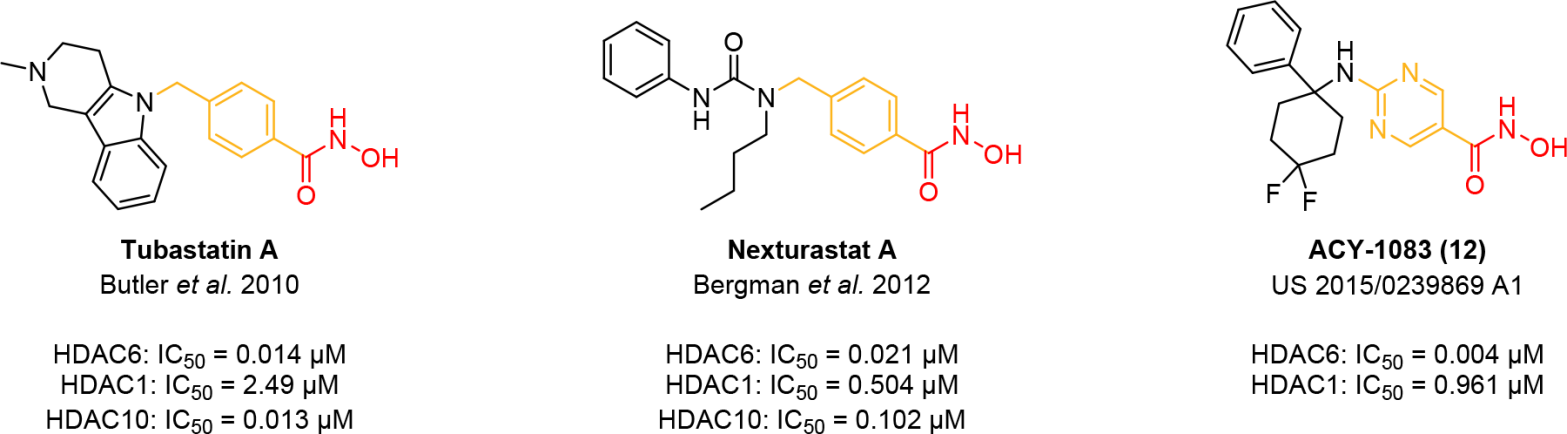
Structures of selected, commercially available hydroxamate-based HDAC6 inhibitors.^189,192,193^ Substances are each labeled with their trade name, IC_50_ values or pIC_50_ values (see Supporting Information Tables S1 and S2) are taken from the following references which were selected according to their comparable assay settings: [Tubastatin A] HDAC1/6: Mackwitz et al. 2018,^194^ HDAC10_TR‑FRET_: Géraldy et al. 2019;^108^ [Nexturastat A] HDAC1/6: Pflieger et al. 2024,^195^ HDAC10_TR‑FRET_: Géraldy et al. 2019;^108^ [ACY1083] HDAC1/6: Mazitschek et al. 2015.^193^ However, reported isoform profile data still result from different assay conditions and should therefore be interpreted with caution.

The structure-based discovery of tubastatin A served as a foundational breakthrough, catalyzing the development of numerous HDAC6 inhibitors over the following decade. These inhibitors commonly adopted a T-shaped architecture, featuring a bulky or branched cap group, a benzyl or aminophenyl linker, and a hydroxamate ZBG. Notable examples include nexturastat A and ACY-1083 (**12**; Figure 10), both widely used HDAC6 tool compounds derived from the tubastatin A pharmacophore model.

A major milestone in the pursuit of truly selective HDAC6 inhibitors was reached in 2016, when Hai and Christianson,^77^ in parallel with Matthias and coworkers,^190^ reported the first cocrystal structures of the CD2 of *Homo sapiens* HDAC6 and both CD1 and CD2 of *Danio rerio* HDAC6. These structural insights revealed several key features of HDAC6 CD2: (1) a unique serine gatekeeper residue (S531) located on the L2 loop, (2) the potential for an alternative monodentate Zn^2+^-binding mode, and (3) a rigid L1 loop that forms a hydrophobic pocket defined by residues H463, P464, F583, and L712.^77,88,190,191^ This L1 loop pocket plays a critical role in accommodating the cap groups of typical T-shaped HDAC6 inhibitors.

Although tubastatin A and nexturastat A have been widely used as “selective” HDAC6 tool compounds for several years, their true selectivity profiles have come into question. Notably, treatment with these compounds at low micromolar or in the case of nexturastat A even submicromolar concentrations induced hyperacetylation of histone H3, suggesting off-target effects on class I HDACs.^195,196^ Subsequent studies using biochemical assays revealed that their selectivity over HDAC1–3 was significantly lower than initially reported.^194,195,197^ Interestingly, systematic studies show that these off-target effects against class I HDAC enzymes are probably the cause for the antiproliferative effects of nonselective HDAC6 inhibitors such as ricolinostat (see Figure 2C) and tubastatin A.^135,198^ Furthermore, Miller and coworkers employed state-of-the-art TR-FRET- and BRET-based assays (see section "Novel Assay Formats to Investigate the Polyamine Deacetylase HDAC10") and demonstrated that tubastatin A is, in fact, a dual HDAC6/10 inhibitor.^108^ Combined with the well-documented liabilities of hydroxamic acids, such as mutagenicity, genotoxicity, and metabolic instability,^199^ this observation marked a turning point, signaling the decline of hydroxamic acids as viable scaffolds for truly selective HDAC6 inhibitors.

Consequently, to avoid the selectivity issues, mutagenicity, genotoxicity associated with hydroxamic acids, many groups from industry and academia embarked on the discovery of alternative ZBGs including carboxylic acids, mercaptoacetamides, fluoroalkyloxadiazoles, and ethylhydrazides.^200^ Unfortunately, most attempts showed limited success. However, one remarkable success story was the discovery of the 2-(difluoromethyl)-1,3,4-oxadiazole (DFMO) group.^201^ The DFMO moiety was initially reported as a ZBG specifically targeting HDAC6 in a 2017 patent filed by Chong Kun Dang Pharmaceutical Corp.^202^ Although it has been commonly mentioned in patent literature, its presence in academic research articles remained limited for several years.

In 2021, Onishi et al. reported that T-518 (**13**), a DFMO-based selective HDAC6 inhibitor (Figure 11A), exhibited therapeutic promise for treating Alzheimer’s disease and tauopathy in mice following oral administration.^104^ The compound’s high selectivity for HDAC6 was validated through both biochemical assays and cellular studies. Additionally, **13** demonstrated favorable pharmacokinetic characteristics and effective brain penetration. Notably, Onishi et al. reported two IC_50_ values for **13** at HDAC6: 0.036 μM without preincubation and 0.0046 μM after 60 min of preincubation, suggesting that **13** is a slow-binding HDAC6 inhibitor.^104^

**Figure 11:**
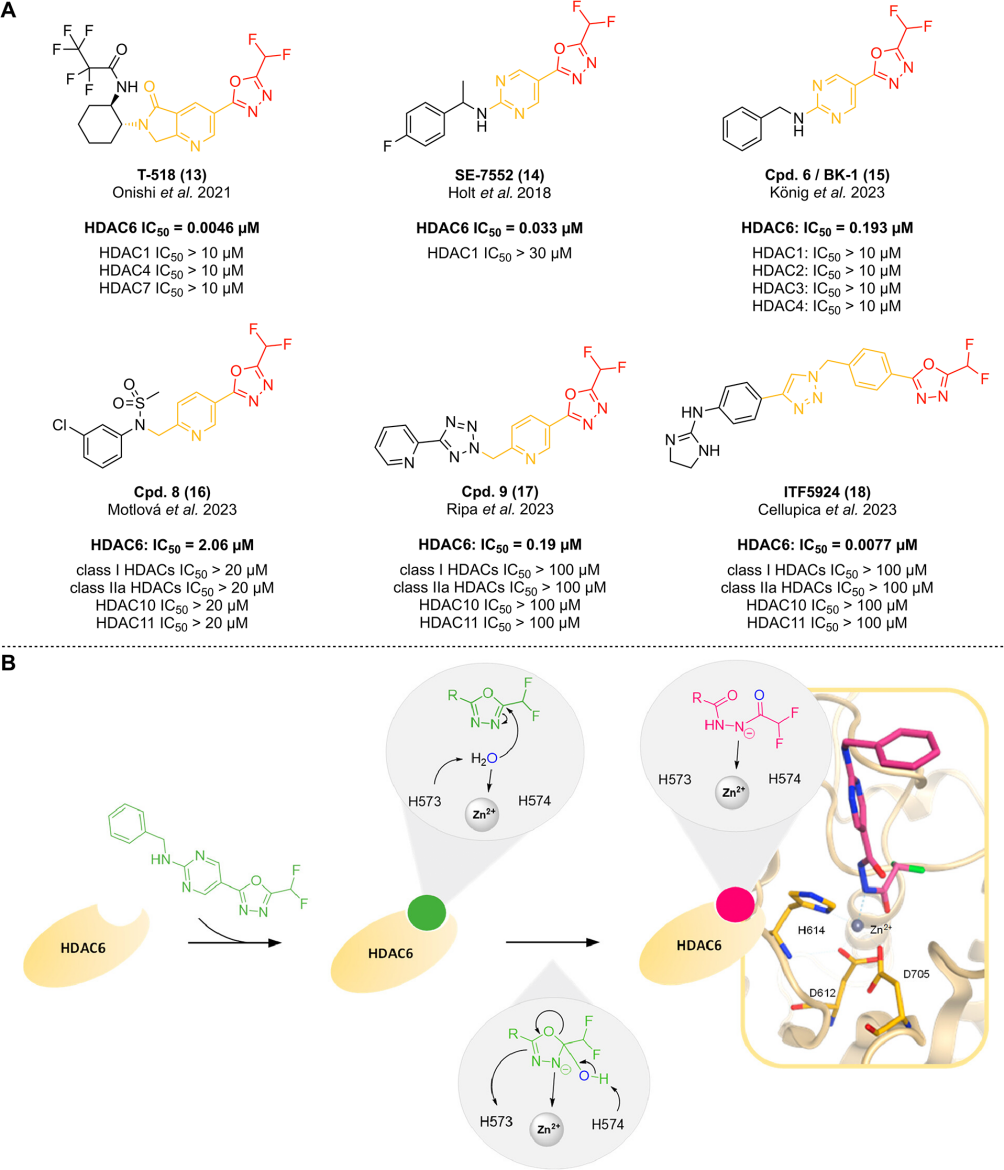
**A)** Structures of selected, commercially available DFMO-based selective HDAC6 inhibitors.^104,119,120,203,204^ Substances are each labeled with their trade name or its designation used in the original publication. Isoform profile data are taken from the respective reference listed beneath the structure except for compound SE-7552 (**14**) where IC_50_ values were taken from patent WO 2018/165520 A1.^205^ **B)** Schematic representation of the HDAC6-catalyzed ring-opening reaction of inhibitor **15**. Right: cocrystal structure of the oxadiazole-derived difluoroacetylhydrazide and HDAC6 (PDB ID: 8GD4). Reprinted with permission from König, B.; Hansen, F. K. *ACS Pharmacol. Transl. Sci.* **2024**, 7 (3), 899–903.^201^ Copyright © 2024 American Chemical Society.

Interestingly, the DFMO derivative SE-7552 (**14**, Figure 11A) was first introduced in a conference abstract in 2018 without disclosing its structure. The authors described **14** as a selective, nonhydroxamate HDAC6 inhibitor, capable of suppressing multiple myeloma *in vivo* and report an IC_50_ value of 0.033 μM at HDAC6 and “greater than 850-fold selectivity versus all other known HDAC isozymes” as well as some pharmacokinetic parameters of **14**.^206^ However, the structure of **14** was only disclosed in 2022 when Cone and coworkers investigated tubastatin A (Figure 10) and **14**.^203^ They employed **14** as a selective HDAC6 inhibitor to counteract leptin resistance in obesity, demonstrating its antiobesity effects in diet-induced obese mice.

Building on these developments, the research groups of Hansen, Gütschow, and Bendas successfully incorporated the DFMO functional group into proteolysis-targeting chimeras (PROTACs) aimed at selectively degrading HDAC6.^207^ Despite these advances, the precise mechanism through which DFMO compounds inhibit or facilitate the degradation of HDAC6 remained enigmatic until 2022:

In a conference abstract, the Christianson group, in collaboration with the Hansen group, reported that the DFMO-based Cpd. 6/BK-1 (**15**; Figure 11A) underwent an enzyme-mediated ring-opening transformation.^208^ This reaction yielded an acylhydrazide, which was cocrystallized with HDAC6, adopting an extended conformation within its active site. Building on their earlier conference presentation, the Christianson and Hansen groups published in 2023 comprehensive crystallographic and mechanistic data confirming that the DFMO warhead undergoes an enzyme-catalyzed ring-opening reaction.^119^ This transformation produces a deprotonated difluoroacetylhydrazide, which serves as the active inhibitory species (Figure 11B). The potent, anionic coordination of this species to the catalytic zinc ion, combined with the fitting of its difluoromethyl group into the P571 pocket of HDAC6’s CD2 domain, results in essentially irreversible enzyme inhibition. The tight-binding nature of this interaction was further validated through *Jump-Dilution* experiments and dialysis assays. Collectively, these findings provide strong support that DFMO compounds function as mechanism-based HDAC6 inhibitors with slow- and tight-binding kinetics.^119^

Almost in parallel, groups from industry also reported the enzyme-catalyzed ring-opening reaction of DFMOs (e.g., Cpd. 8 (**16**; Motlová et al. 2023),^209^ Cpd. 9 (**17**; Ripa et al. 2023),^120^ and ITF5924 (**18**; Cellupica et al. 2023),^204^ for structures see Figure 11A). However, they initially cocrystallized the corresponding hydrazide derivative which was presumably obtained by a second enzyme-catalyzed hydrolysis of the difluoroacetylhydrazide intermediate. Later, Vergani and coworkers cocrystallized a difluoroacetylhydrazide:HDAC6 CD2 complex and proposed two HDAC6-catalyzed DFMO hydrolysis mechanisms, differing in whether the difluoroacetylhydrazide intermediate dissociates from HDAC6 after the initial hydrolysis step.^210^ The authors also assign the tight-binding properties of DFMO-based inhibitors to the resulting difluoroacetylhydrazide species. Taken together, there is now clear evidence that the conversion of DFMOs to the corresponding difluoroacetylhydrazides and hydrazides is responsible for their highly selective and efficient HDAC6 inhibition.

All DFMO-based selective HDAC6 inhibitors summarized in Figure 11A are commercially available and can serve as effective tool compounds for studying the functional effects of HDAC6 CD2 inhibition. Among these, **17** is the best-characterized inhibitor based on current data.^120^ It exhibits highly selective HDAC6 inhibition both biochemically (HDAC6 IC_50_ = 0.19 μM; IC_50_ > 100 μM for all other Zn^2+^-dependent isoforms) and at the cellular level (acetyl α-tubulin vs acetyl histone H3 levels).^120^ Additionally, Ripa et al. observed a remarkable IC_50_-shift for **17** depending on its preincubation time at HDAC6: IC_50_ = 0.19 μM (5 min) vs 0.027 μM (30 min) vs 0.0011 μM (20 h). These findings are in good agreement with those of other groups (Onishi et al. (**13**)^104^ and König and Watson et al. (**15**)^119^ report similar slow-binding properties for their DFMO derivatives) and underscore the unparalleled, mechanism-based selectivity of DFMO-based HDACi toward HDAC6. Importantly, **17** also displayed high specificity for HDAC6 in a broad selectivity panel covering 92 enzymes, receptors, ion channels, and transporters. It also demonstrated a favorable *in vitro* safety profile, with no detectable activity on key cardiovascular ion channels and no genotoxicity in micronucleus assays.^120^ Furthermore, **17** showed high oral bioavailability (*F* ∼ 100% in mouse and rat) as well as low *in vivo* clearance (e.g., rat: CL (**17**) = 3.2 mL/min/kg vs CL (**12**, ACY-1083) = 180 mL/min/kg) resulting in a remarkably long plasma half-life (*t*_1/2_ = 10 h; p.o., rat), and is thus a valuable tool for *in vitro* and *in vivo* studies of HDAC6 function. Of note, Ripa et al. reported that DFMOs remain stable at neutral pH (7), but undergo chemical degradation under acidic or basic conditions. This instability may pose a limitation for compound **17** and for DFMOs in general as tool compounds. Nevertheless, their stability under neutral conditions suggests that appropriate formulation strategies could mitigate these challenges, as has been achieved with other acid-sensitive blockbuster drugs such as proton pump inhibitors. Overall, **17** is a highly selective HDAC6 inhibitor that is thoroughly characterized both *in vitro* and *in vivo*, with comprehensive evaluation of cardiovascular safety, genotoxicity, and off-target activity. Therefore, **17** is our recommended tool compound for studying HDAC6 inhibition (also see Table 2).

### Class IIb HDACs – HDAC10

The discovery that HDAC10 functions as a polyamine deacetylase^83,84^ along with the development of improved HDAC10 inhibition assays,^108,109^ laid the groundwork for the identification of truly selective HDAC10 inhibitors (see section “Novel Assay Formats to Investigate the Polyamine Deacetylase HDAC10”). In 2019, Miller and coworkers reported a series of ring-opened tubastatin A analogs exhibiting up to 40-fold selectivity for HDAC10 over HDAC6.^108^ These compounds retained the benzyl linker and hydroxamic acid ZBG of tubastatin A, while incorporating an indole-based cap group with a basic tertiary amine designed to interact with a gatekeeper glutamate residue (e.g., Cpd. 28 (**19**), see Figure 12). However, this scaffold remained unsuitable for the development of a chemical probe for HDAC10 due to its residual HDAC6 inhibitory properties.^108^

**Figure 12:**
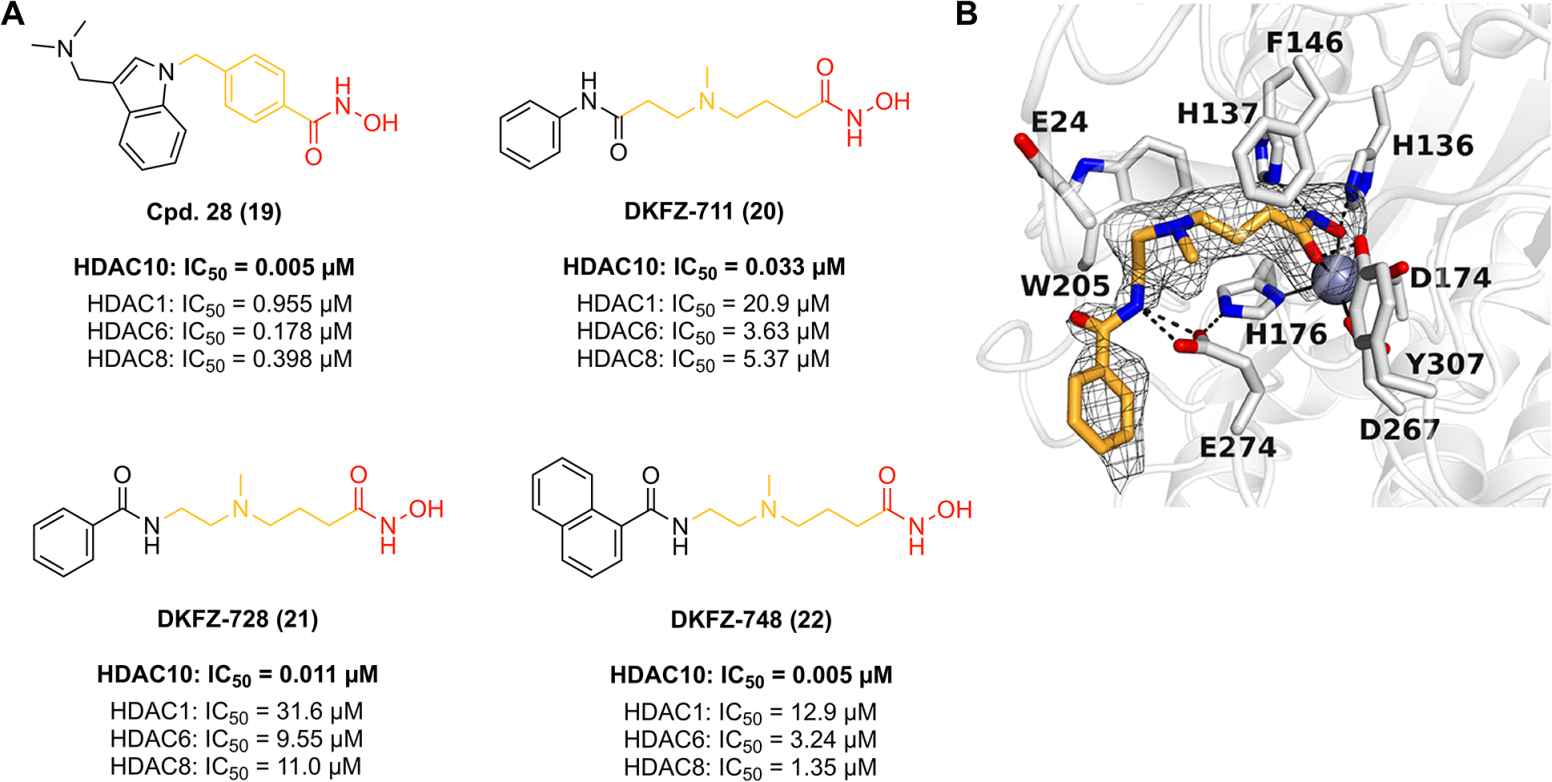
A) Structures of the ring-opened tubastatin A analog Cpd. 28 (**19**)^108,211^ as well as the structures of the HDAC10 selective Aza-SAHA analogs DKFZ-711 (**20**), DKFZ-728 (**21**), and DKFZ-748 (**22**) published by Steimbach et al.^196^ Substances are each labeled with their trade name or designation used in the original publication. Isoform profile data are taken from Géraldy et al. (**19**) or Steimbach et al. (**20**–**22**). Reported pIC_50_ values were converted to IC_50_ values (see Supporting Information Tables S1 and S2).^108,196^ **B)** Co-crystal structure of **21** bound to HDAC10. HDAC10 backbone is shown as light gray cartoon, side chain amino acids that show specific interactions with the ligand (light gray) as well as the ligand (orange) are depicted as sticks. The simulated annealing omit map of **21** is contoured at 2.2σ. The catalytic Zn^2+^-ion is shown as a gray sphere, metal coordination interactions are shown as solid black lines, hydrogen bonds are represented as dashed black lines. Reprinted with permission from Steimbach et al. *J. Am. Chem. Soc.* **2022**, 144 (41), 18861–18875. Copyright © 2022 American Chemical Society.

A breakthrough was achieved by the same group through a systematic “aza-scan,” in which an methylamino group was inserted into the hexyl linker of the FDA-approved pan-HDAC inhibitor vorinostat (SAHA).^196^ This modification was inspired by the unique substrate specificity of HDAC10 for acetylated polyamines such as *N* ^8^-acetyl spermidine, *N*-acetyl putrescine, and *N-*acetyl cadaverine. The initial result of this strategy was the development of the direct Aza-SAHA analog DKFZ-711 (**20**, Figure 12A). This simple C-to-N substitution transformed vorinostat from a nonselective HDAC inhibitor into a highly specific HDAC10 inhibitor, demonstrating over 100-fold selectivity for HDAC10 over HDAC6.^196^ Further optimization involved inverting the anilide moiety in **20** to a benzamide, resulting in DKFZ-728 (**21**, Figure 12A). This compound exhibited enhanced HDAC10 inhibitory potency and significantly reduced off-target activity, achieving approximately 890-fold selectivity over HDAC6 and 2900-fold over HDAC1.^196^ Crystal structures were solved for both the anilide **20** and the benzamide **21** in complex with a “humanized” form of *Danio rerio* HDAC10 (see Figure 12B), engineered with two mutations near the binding site to better mimic the human enzyme. Both compounds showed similar binding interactions with HDAC10, including a weak cation−π interaction with W205. However, unlike **20**, **21** also formed a long-range electrostatic interaction with the gatekeeper residue E274 (Figure 12B), likely contributing to its enhanced selectivity and potency.^196^

The systematic variation of the cap group of **21** generated the naphthamide derivative DKFZ-748 (**22**, Figure 12A). This compound demonstrated a cellular IC_50_ for HDAC10 of 22 nM with more >500-fold selectivity over other isoforms. The exceptional selectivity profile was further validated by chemoproteomic profiling. It was confirmed that **22** inhibits spermidine deacetylation in cells with no off-target hyperacetylation.^196^ Furthermore, **22** was evaluated in a polyamine-limited *in vitro* tumor model, where pharmacological inhibition of HDAC10 by **22** phenocopied its genetic knockout, as evidenced by dose-dependent suppression of *N* ^8^-acetyl spermidine-mediated growth rescue.^196^ **22** also demonstrated low cytotoxicity across a panel of cancer cell lines, highlighting its suitability as a tool compound for investigating HDAC10 function in noncancerous disease contexts. Collectively, these findings establish **22** as the most comprehensively characterized selective HDAC10 inhibitor to date. Based on the available data and its commercial accessibility, **22** is our recommended tool compound for HDAC10 research (see Table 2).

### Class IV HDACs – HDAC11

Although HDAC11 was first reported in 2002, it is still one of the less-studied HDAC isoforms and its biological function remains largely unexplored.^215,216^ In practice, HDAC11 research is still complicated due to the absence of an HDAC11 crystal structure. However, several new *in vitro* HDAC11 assays have been reported in recent years (see section “*In Vitro* Profiling of HDAC Inhibition”), which now enable the systematic development of potent and selective HDAC11 inhibitors and thus the development of chemical probes for HDAC11.

SIS17 (**23**) was first reported alongside a series of other alkyl-hydrazide HDACi by Son et al. in 2019 (see Figure 13).^112^ According to *in vitro* HDAC assays, **23** exhibits at least 200-fold selectivity over other HDAC isoforms as well as robust antineoplastic effects against AML cell lines.^112,213^ Furthermore, cellular target engagement of **23** at HDAC11 was confirmed by Western blot using dose-dependent acylation of SHMT2 as readout.^112^ Additionally, treatment with **23** did not lead to increased levels of acetyl α-tubulin (HDAC6) or acetyl histone H3 (class I HDACs) indicating HDAC11 selectivity in a cellular setting.^112^ In 2023, Ho et al. published a series of trapoxin A analogues.^214^ Their hit compound TD034 (**24**) was found to be a potent (IC_50_ = 0.005 μM), competitive, and reversible inhibitor of HDAC11 which showed notable selectivity (>5000-fold) over HDAC1, 4, 6, and 8 in *in vitro* HDAC assays. Additionally, the authors determined the HDAC11 affinity of **24** and calculated a *K*_i,HDAC11_ of 1.5 nM. The observed selectivity of **24** for HDAC11 was further confirmed in a cellular setting using Western blot: treatment of HEK293T cells with increasing concentrations of **24** resulted in increasingly elevated SHMT2 acylation levels but did not lead to higher levels of acetyl histone H3 (class I HDACs), acetyl α-tubulin (HDAC6), or acetyl p53 (HDAC1–3, HDAC8).^214^ In the same year, Bai et al. reported the brain-permeable HDAC11 inhibitor PB94 (**25**) which ameliorated neuropathic pain in a mouse model.^212^ With an IC_50_ value of 108 nM at HDAC11, **25** is less potent than **24** and shows only >40-fold selectivity over other HDAC isoforms.^212^ Notably, HDAC11 selectivity was not confirmed in a cellular setting since off-target effects at other HDAC isoforms (e.g., HDAC6 or class I HDACs) have not been tested. However, the authors provide basic pharmacokinetic characteristics as well as data concerning the metabolic stability and toxicity of **25** in mice. Additionally, PET imaging studies of [^11^C]­PB94 in mice further confirmed the good brain permeability of **25** (brain/plasma ratio: 1.5–2.3) and gave valuable insights into its biodistribution in selected peripheral organs.^212^ Overall, **25** was well tolerated up to 200 mg/kg (NOAEL; mice). However, **25** showed only moderate metabolic stability, notable CYP inhibition at 10 μM, and low oral bioavailability (*F* = 11.2%; 10 mg/kg (p.o.); mouse).^212^

**Figure 13:**
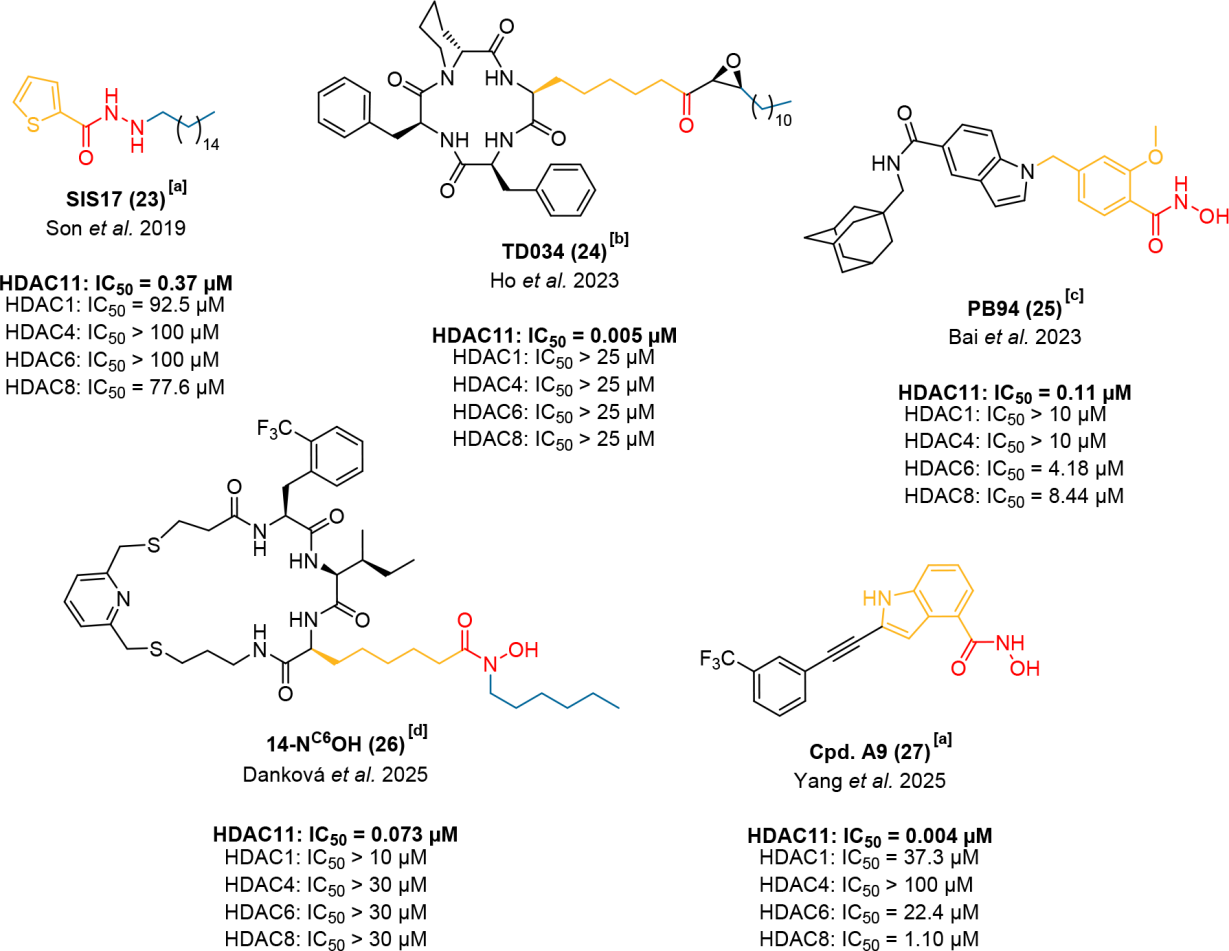
Structures of selected, commercially available HDAC11 inhibitors.^112,133,212–214^ IC_50_ values are taken from the following references: [a] Yang et al. 2025;^213^ [b] Ho et al. 2023;^214^ [c] Bai et al. 2023;^212^ [d] Danková et al. 2025.^133^ Notably, reported isoform profile data result from different assay conditions and should therefore be interpreted with caution.

More recently, the Olsen and Heinis laboratories introduced 14-N^C6^OH (**26**), a potent (IC_50_ = 0.073 μM) macrocyclic HDAC11 inhibitor bearing a novel *N*-alkylated hydroxamic acid ZBG.^133^ Importantly, this study provides *K*_M_ values for all enzyme/substrate pairs used in the *in vitro* HDAC assays, which is still uncommon in literature. Thus, the authors are able to report the HDAC11 affinity of **26** with a *K*_i,HDAC11_ of 40 nM. Further, Danková et al. confirmed HDAC11 target engagement of **26** via a pulldown assay using two specifically designed, biotin-labeled high-affinity probes. Additionally, they investigated the cellular off-target effects of **26** against class I HDACs using Western blot. Notably, treatment of HEK293T cells with **26** (5 h, 5 μM) did not lead to a measurable increase in global histone acetylation or at the specific acetylation sites H3K18ac, H3K27ac, and H3K36ac indicating no inhibition of class I HDACs up to 5 μM in a cellular environment. However, other acetylation sites such as acetyl α-tubulin (HDAC6) or acetyl SMC3 (HDAC8) were not investigated. Finally, **26** was identified as a highly cell permeating compound using a chloroalkane penetration assay (CAPA).^133^

Earlier this year (2025), Yang et al. published an interesting series of L-shaped HDAC11 inhibitors, including Cpd. A9 (**25**).^213^ **25** is a potent and selective HDAC11 inhibitor that demonstrated moderate antitumor effects in several AML cell lines, as well as robust antitumor efficacy, both alone and in combination with cytarabine, in a mouse xenograft AML model.^213^ Notably, treatment of the U937 AML cell line with **25** led to the same antitumor effects as HDAC11 knockdown in U937 cells confirming that **25** related antitumor effects result from selective HDAC11 inhibition. Besides this, **25** possesses good metabolic stability (*t*_1/2_ = 8.95 h; 50 mg/kg (i.p.); mouse) and acceptable bioavailability (*F* = 32%; 50 mg/kg (i.p.); mouse) and was well tolerated *in vivo* (50 mg/kg; duration of treatment ≥ 14 d; mouse).

In summary, with compounds **23**, **24**, **26**, and **27** there are now several HDAC11 selective inhibitors available to investigate HDAC11 function in a cellular environment. Given its superior HDAC11 affinity as well as selectivity over other HDAC isoforms, the trapoxin A-derived inhibitor **24** is the most obvious choice for *in vitro* studies which are limited to the cellular environment. For *in vivo* studies, **27** seems to be better suited as a tool compound given its overall more extensive data set compared to **24**. However, since **27** shows only mediocre bioavailability after i.p. application and the current data set lacks information regarding its off-target toxicity (e.g., *h*ERG, CYPs, etc.) as well as overall toxicity *in vivo* (e.g., NOAEL), there are still some restrictions for its use as a tool compound which warrant further research. Given these issues, it would also be interesting to investigate if compounds **23**, **24**, or **26** are better suited for *in vivo* applications than 27 ( see Table 2).

## Conclusion

The use of HDACi as chemical probes has significantly advanced our understanding of chromatin regulation and epigenetic control. However, progress in the field has been slowed by the premature application of insufficiently characterized inhibitors: In the past, oversimplified biochemical assays often overstated isoform selectivity, leading to the adoption of compounds such as RGFP966 (**6**) or tubastatin A as “specific” probes. However, later studies revealed that RGFP966 (**6**) potently inhibits HDAC1–3 rather than HDAC3 alone, while tubastatin A displays substantial HDAC10 activity in addition to HDAC6. These cases illustrate how flawed tool compounds can misdirect research and underscore the necessity for rigorous probe validation.

In this context, isoform-specific features such as subpocket architecture, substrate preferences, binding kinetics, and corepressor complex selectivity must be carefully considered. Recent advances in structural biology and enzymology as well as novel, more sophisticated *in vitro* assay systems provide a refined framework for assessing HDAC selectivity and thus enable the comprehensive characterization of potential chemical probes. That way, several new, highly specific tool compounds have been identified such as the CoREST-selective inhibitors Rodin-A (**10**) and TNG260 (**11**), as well as the HDAC10-specific inhibitor DKFZ-748 (**22**), which now represent more reliable choices for mechanistic studies.

On the other hand, there is also value in the thorough (re)­evaluation of well-established inhibitors such as tacedinaline or entinostat to unravel their complex biological effects. However, although future efforts should focus on the design and validation of tool compounds with clearly defined selectivity, it is equally important to exclude flawed or poorly profiled inhibitors from mechanistic investigations, to avoid perpetuating misleading conclusions. Furthermore, to ensure the reliability of biological insights, multiple validated probes and orthogonal approaches such as knockdown and/or -out experiments should be employed in parallel.

In conclusion, the careful and critical use of HDACi as chemical probes is essential for disentangling isoform-specific biology and advancing therapeutic discovery. By learning from past mistakes and emphasizing rigor in probe development, the field is well positioned to deliver next-generation HDAC modulators that are not only powerful research tools but also promising candidates for treating HDAC-related diseases such as cancer and neurodegeneration.

## Supplementary Material



## Notes

Transformation of pIC_50_ values to IC_50_ values in some cases; documentation in Tables S1 and S2 (PDF)

## Abbreviations

ADP: adenosine diphosphate
AMC: 7-amino-4-methylcoumarin
AUC: area under the plasma concentration curve
BAX: bcl-2-like protein 4
BRET: bioluminescence resonance energy transfer
CAF-1: chromatin assembly factor 1
CD: catalytic domain
CL: clearance
*C*_max_: maximum plasma concentration
CoREST: repressor element-1 silencing transcription factor corepressor
Cpd.: compound
CYPs: cytochrome P450 enzymes
DFMO: 2-(difluoromethyl)-1,3,4-oxadiazole
Diss. *t*_1/2_: dissociative half-life
DNA: deoxyribonucleic acid
DNMT: DNA methyltransferase
ERRα: estrogen-related receptor alpha
*F*: bioavailability
FDA: Food and Drug Administration
FPU: foot-pocket unit
HDAC(s): histone deacetylase(s)
HDACi: histone deacetylase inhibitor
*h*ERG: human Ether-à-go-go-Related Gene
HIV: human immunodeficiency virus
HPLC: high performance liquid chromatography
IC_50_: half maximal inhibitory concentration
i.p.: intraperitoneal application
LC-MS: liquid chromatography–mass spectrometry
MAL: *tert*-butyl (*S*)-{6-acetamido-1-[(4-methyl-2-oxo-2*H*-chromen-7-yl)­amino]-1-oxohexan-2-yl}­carbamate
MEF2: myocyte enhancer factor 2
Mesna: 2-mercaptoethanesulfonic acid sodium salt
MiDAC: mitotic deacetylase complex
NAD: nicotinamide adenine dinucleotide
NCoR1: nuclear receptor corepressor 1
NCoR2: nuclear receptor corepressor 2 or: silencing mediator of retinoic acid and thyroid hormone receptor, see SMRT
NDA: naphthalene-2,3-dialdehyde
NES: nuclear export sequence
NLS: nuclear localization sequence
NMPA: national medical products administration
NOAEL: no observed adverse effect level
NSCLC: nonsmall cell lung cancer
NuRD: Mi-2/nucleosome remodeling deacetylase
PET: positron emission tomography; p.o., peroral application
PROTAC(s): proteolysis-targeting chimera(s)
SAR(s): structure activity relationship(s)
s.c.: subcutaneous application
SHMT2: myristoyl-serine hydroxymethyl transferase 2
Sin3A: switch-independent 3A
siRNA: small-interfering ribonucleic acid
SMC3: structural maintenance of chromosomes protein 3
SMRT: silencing mediator of retinoic acid and thyroid hormone receptor
STK11: serine/threonine kinase 11
*T*_1/2_: drug-target residence time
*t*_1/2_: plasma half-life
TFA: trifluoroacetic acid
TNFα: tumor necrosis factor α
TR-FRET: time-resolved fluorescence resonance energy transfer
Vd_ss_: volume of distribution
WT: wild-type
ZBG(s): zinc binding group(s)
ZMAL: benzyl (*S*)-{6-acetamido-1-[(4-methyl-2-oxo-2*H*-chromen-7-yl)­amino]-1-oxohexan-2-yl}­carbamate
ZnF: zinc finger.
