# Supplementary material for: Recommended Tool Compounds: Isoform- and Class-Specific Histone Deacetylase Inhibitors
Source: ACS Pharmacol Transl Sci. 2026 Feb 20;9(3):462–89. doi: 10.1021/acsptsci.5c00619 (PMC12993782; doi:10.1021/acsptsci.5c00619)
Supplement: Supplementary file 1 [file pt5c00619_si_001.pdf]

Supporting Information

Recommended Tool Compounds: Isoform- and Class-specific Histone Deacetylase Inhibitors

Linda Schäker-Hübner[a] and Finn K. Hansen[a]*

[a]Pharmaceutical Institute, University of Bonn, An der Immenburg 4, 53121 Bonn, Germany.

* Corresponding Author: Linda Schäker-Hübner, Email: l.schaeker@uni-bonn.de

Table of Contents

SUPPLEMENTAL TABLES **S2**

Table S1. Originally reported pIC_{50} values of selected HDAC6 and HDAC10 inhibitors. S2

Table S2. IC_{50} values of selected HDAC inhibitors calculated from pIC_{50} values (Table S1).‡ S2

SUPPLEMENTAL TABLES

Table S1. Originally reported pIC_{50} values of selected HDAC6 and HDAC10 inhibitors.

	**pIC_{50} [M]**					
	**HDAC1**	**HDAC2**	**HDAC3**	**HDAC6**	**HDAC8**	**HDAC10**
Tubastatin A[a]						7.90
Nexturastat A[a]						6.99
Cpd. 28[a]	6.02	5.21	6.23	6.75	6.40	8.28
DKFZ-711[b]	4.68	4.13	4.11	5.44	5.27	7.48
DKFZ-728[b]	4.50	4.07	< 4.00	5.02	4.96	7.97
DKFZ-748[b]	4.89	4.29	< 4.00	5.49	5.87	8.29

[a]Data taken from Géraldy, M. *et al. J. Med. Chem.* **2019**, *62* (9), 4426–4443. [b]Steimbach, R. R. *et al. J. Am. Chem. Soc.* **2022**, *144* (41), 18861–18875.

Table S2. IC_{50} values of selected HDAC inhibitors calculated from pIC_{50} values (Table S1).‡

	**IC_{50} [µM]**					
	**HDAC1**	**HDAC2**	**HDAC3**	**HDAC6**	**HDAC8**	**HDAC10**
Tubastatin A						0.0126
Nexturastat A						0.102
Cpd. 28	0.955	6.17	0.589	0.178	0.398	0.00525
DKFZ-711	20.9	74.1	77.6	3.63	5.37	0.0331
DKFZ-728	31.6	85.1	> 100	9.55	11.0	0.0107
DKFZ-748	12.9	51.3	> 100	3.24	1.35	0.00513

‡IC_{50} values were calculated from the respective pIC_{50} values (Table S2) using the following equation:

$$IC_{50} = 10^{(-pIC_{50})} \times 10^{6}$$
